# Response of seed yield and biochemical traits of *Eruca sativa* Mill. to drought stress in a collection study

**DOI:** 10.1038/s41598-023-38028-6

**Published:** 2023-07-10

**Authors:** Sharifeh Nikzad, Seyed Ali Mohammad Mirmohammady Maibody, Mohammad Hossein Ehtemam, Pooran Golkar, Seyed Abolghasem Mohammadi

**Affiliations:** 1 Department of Agronomy and Plant Breeding, College of Agriculture, Isfahan University of Technology, Isfahan, 84156 83111 Iran; 2 Department of Natural Resources, Isfahan University of Technology, Isfahan, 84156 83111 Iran; 3 Department of Plant Breeding and Biotechnology, Faculty of Agriculture, University of Tabriz, Tabriz, Iran

**Keywords:** Biochemistry, Plant sciences

## Abstract

Drought tolerance is a complex trait in plants that involves different biochemical mechanisms. During two years of study (2019–2020), the responses of 64 arugula genotypes to drought stress were evaluated in a randomized complete block design with three replications under field conditions. Several metabolic traits were evaluated, i.e. relative water content, photosynthetic pigments (chlorophyll and carotenoids), proline, malondialdehyde, enzymatic antioxidants (catalase, ascorbate peroxidase, and peroxidase), total phenolic and flavonoid contents and seed yield. On average, the drought stress significantly increased the proline content (24%), catalase (42%), peroxidase (60%) and malondialdehyde activities (116%) over the two years of study. As a result of the drought stress, the seed yield (18%), relative water content (19.5%) and amount of photosynthetic pigments (chlorophyll and carotenoids) dropped significantly. However, the total phenolic and flavonoid contents showed no significant changes. Under drought stress, the highest seed yields were seen in the G_50_, G_57_, G_54_, G_55_ and G_60_ genotypes, while the lowest value was observed in the G_16_ genotype (94 g plant^−1^). According to the findings, when compared to the drought-sensitive genotypes, the drought-tolerant arugula genotypes were marked with higher levels of proline accumulation and antioxidant enzyme activity. Correlation analysis indicated the positive effects of peroxidase, catalase and proline on seed yield under drought conditions. These traits can be considered for the selection of drought-tolerant genotypes in breeding programs.

## Introduction

Arugula (*Eruca sativa* Mill.) (Brassicaceae) is an annual, herbaceous oil crop and has a broad range of valuable industrial and pharmaceutical applications^1^. According to the available literature, arugula originated in the Mediterranean region. Today, arugula is widely cultivated in different regions, such as North Africa, Eastern Asia (Pakistan, Afghanistan, and India), and the Middle East (Iraq, Iran, and Syria)^2^. The extracted arugula oil can be used for manufacturing biodiesel and various other industrial products^3^.

The seeds and leaves of the plant have been used in different medicinal and industrial applications^1^. Arugula is considered a source of several phytochemicals with health-promoting properties (e.g. fibers, carotenoids, flavonoids, glucosinolates and vitamin C)^4^. The plant reportedly has different medicinal properties. It is considered a digestive, astringent, diuretic, tonic, emollient, depurative, laxative, and stimulant agent^1^. Moreover, the spicy taste of the buds and leaves of this plant have broadened its use in foods and salads^1,2^. Nowadays, arugula is being widely cultivated around the world due to its importance in different fields, such as agriculture, medicine and various industries^5^.

Drought stress has been identified as one of the most critical environmental stresses, and leads to harmful effects on crop performance by restricting plant yield and productivity^6^. Drought-prone environments adversely affect plant yield and quality^7^. Certain factors including genotype, species, developmental stage, stress duration and severity can impact plant response to water limitation^8^. Different physiological and molecular processes are involved when plants respond to the deleterious effects of drought stress through scavenging^7^, such as the accumulation of various osmolytes and secondary metabolites (SMs), the increase of different enzymatic and non-enzymatic antioxidant activities, and the activation of certain mechanisms to compensate the water content loss in leaves^9^.

Under environmental stresses, such as the drought stress, an increasing trend has been observed in the production of certain reactive oxygen species (ROS), such as hydrogen peroxide (H_2_O_2_), superoxide (O_2_^·^), hydroxyl (^•^OH) and alkoxy radical (RO^·^)^10^. To scavenge the deleterious effects of ROS, the enzymatic and non-enzymatic defense systems have evolved under environmental stresses^10^. Therefore, certain enzymes such as catalase (CAT), peroxidase (POX), ascorbate peroxidase (APX) and glutathione reductase (GR) have been activated in the scavenging system to minimize the oxidative damages to plants^9,11^. On the other hand, the different SMs that act as non-enzymatic antioxidants, such as leaf pigments (e.g. carotenoids), and different phenolic compounds (e.g. phenolics, flavonoids and anthocyanins) have a protective role in avoiding ROS generation^12^. The production of SMs by a plant species depends to a great extent on the differential impact of the environmental stress and its severity on the metabolic pathways associated with their biosynthetic pathways^7^. Several studies showed that stressed plants created higher concentrations of SMs, compared to non-stressed plants^13,14^.

A deeper understanding of drought-tolerant mechanisms in plants can be used to select superior genotypes to obtain better productivity under drought stress conditions. The breeding activities that have been carried out on arugula are quite limited. The number of studies that have examined the responses of certain traits of the arugula plant to drought stress are few^15,16^. To the best of our knowledge, there is no information available regarding the effects of drought stress on the seed yield (SY) and the different biochemical traits of arugula.

One of the major goals of plant breeding when faced with climate change is to determine and improve drought-tolerant genotypes. This investigation is a continuation of our previous efforts, and provides further insight regarding the response of different physio-biochemical traits to drought stress in a wide range of arugula germplasm. Therefore, the present study is the first research to assess the effects of drought stress on seed yield and the changes that occur in the physiological and biochemical traits of arugula genotypes. The objectives of the present study were: (1) to investigate the physio-biochemical and antioxidant responses of arugula genotypes to drought stress and their relations with seed yield, and (2) to identify superior arugula genotypes best suited for drought tolerance improvement in arugula germplasm for future programs.

## Results

The analysis of variance showed that the effects of years, genotypes, and environment (non-stress and drought stress) were significant on all traits (Table 1). The year × environment interaction was significant for proline content, SY, Chla, TChl, carotenoids, and CAT (Table 1). With the exception of TPC, TFD, RWC, Chla, and APX, a significant interaction of genotype × environment was detected in all other studied traits (Table 1). The year × genotype interaction was non-significant for Chla, APX, RWC, TPC, and TFD (Table 1). Significant year × genotype and environment × genotype interactions were seen in SY, proline, Chlb, TChl, Car, POX, CAT, MDA and H_2_O_2_ (Table 1). In addition, the year × genotype × environment interaction was significant for SY, proline, Chlb, Car, POX, CAT, and MDA (Table 1).

**Table 1:** Analysis of variance for different traits of 64 arugula genotypes (G) evaluated at two levels of irrigation (I) during the two years (Y) of 2019 and 2020.

S. O. V	DF^I^	SY	RWC	Proline	Chla	Chlb	TChl	Car	POX	APX	CAT	MDA	TPC	TFD	
Y	1	1,264,257**	781.6**	0.013**	0.013**	0.01**	0.41*	0.156**	0.197**	34.91**	0.012**	0.014**	317.67**	21.9**	
I	1	162,436**	34,928**	0.46**	0.191**	0.102**	7.08**	1.025**	1.76**	1063**	1.30**	0.33**	25,827**	147.8**	
Y × I	1	4486**	8.53^ns^	0.0039**	0.004**	0.0006^ns^	1.65**	0.008**	0.0013^ns^	0.46^ns^	0.0003*	0.0002^ns^	6.01^ns^	2.33^ns^	
R (Y × I)	8	63.94^ns^	8.26^ns^	0.00005**	0.0003^ns^	0.0002^ns^	0.07^ns^	0.0003^ns^	0.0005**	0.63**	0.0004^ns^	0.0003**	3.79^ns^	1.01**	
G	63	2519.3**	458.6**	0.0006**	0.004**	0.0015**	0.26**	0.018**	0.004**	7.53**	0.0007**	0.0007**	294.79**	10.75**	
Y × G	63	1748.06**	5.04^ns^	0.00005**	0.001^ns^	0.0008**	0.18**	0.0008**	0.0003**	0.05^ns^	0.0001**	0.0001**	4.48^ns^	0.05^ns^	
I × G	63	621.1**	6.53^ns^	0.00004**	0.0008^ns^	0.0007**	0.15**	0.0015**	0.0003**	0.06^ns^	0.0005**	0.00004**	4.72^ns^	0.05^ns^	
Y × I × G	63	609.38**	4.51^ns^	0.00002**	0.0009^ns^	0.0009**	0.12^ns^	0.0003**	0.0003**	0.085^ns^	0.00004**	0.00002**	4.01^ns^	0.04^ns^	
Residual	503	34.07	22.29	0.00001	0.0009	0.0006	0.111	0.0001	0.0001	0.311	0.00002	0.00001	4.67	0.163	

^I^*DF* degrees of freedom, *SY* seed yield, *RWC* relative water content, *Chla* chlorophyll a, *Chl b* chlorophyll b, *TChl* total chlorophyll, *Car* carotenoids, *POX* peroxidase, *APX* ascorbate peroxidase, *CAT* catalase, *MDA* malondialdehyde, *TPC* total phenolics content, *TFD* total flavonoids.

^ns^Non-significant; * and **: significant at *P* < 0.05 and *P* < 0.01, respectively.

### Effects of drought stress on the studied traits

The investigated traits were compared under two environmental conditions (non-stress and drought stress) (Table 2). As shown by the comparisons of the interaction effects of year × environment, the values obtained for the investigated traits in 2020 were greater than those calculated in 2019. According to the results, the drought stress had a diminishing impact on the photosynthetic pigments (TChl, Car, and Chla) and SY in both years, whereas the levels of the other traits, such as proline content, RWC, MDA, and antioxidant enzyme activity (POX, APX, and CAT) increased significantly (Table 2).

**Table 2:** Mean values obtained for traits studied under non-stress and drought stress conditions in two consecutive years.

Year	Environment	SY^I^ (g plant^−1^)	RWC (%)	Proline (μmol g^−1^ FW)	Chla (mg g^−1^ FW)	Chlb (mg g^−1^ FW)	TChl (mg g^−1^ FW)	Car (mg g^−1^ FW)	POX (µmol min^−1^ mg^−1^ protein)	APX (units mg^−1^ protein)	CAT (units mg^−1^ protein)	MDA (nmol g^−1^ FW)	TPC (mg TAE g^−1^ DW)	TFD (mg QEg^−1^ DW)
2019	Non-stress	^¥^112.3^c^	76.52^a^	0.194^d^	0.124^b^	0.076^a^	0.201^b^	0.337^b^	0.134^d^	4.44^a^	0.106^d^	0.077^d^	28.75^a^	3.76^a^
	Drought stress	88.03^d^	62.79^a^	0.239^b^	0.098^d^	0.051^a^	0.149^d^	0.271^d^	0.232^b^	6.85^a^	0.187^b^	0.17^b^	40.20^a^	4.53^a^
2020	Non-stress	198.3^a^	79.16^a^	0.202^c^	0.141^a^	0.085^a^	0.227^a^	0.384^a^	0.182^c^	5.09^a^	0.116^c^	0.09^c^	30.39^a^	4.13^a^
	Drought stress	164.4^b^	62.83^a^	0.255^a^	0.105^c^	0.064^a^	0.169^c^	0.304^c^	0.275^a^	7.40^a^	0.200^a^	0.19^a^	42.19^a^	5.11^a^

^I^*SY* seed yield per plant, *RWC* relative water content, *Chla* chlorophyll a, *Chlb* chlorophyll b, *TChl* total chlorophyll, *Cars* carotenoids, *POX* peroxidase, *APX* ascorbate peroxidase, *CAT* catalase, *MDA* malondialdehyde, *TPC* total phenolics content, *TFD* total flavonoids.

^¥^ In each column (each trait) the means with similar letters are not significant at *P* < 0.01 by the LSD test. Values are means ± S.E. (replication = 3).

### Seed yield

The means of the studied genotypes showed a significant SY reduction under drought stress conditions (Table 2). A wide range of variation was observed in SY under the non-stress (107.5–185.17 g plant^−1^) and drought stress (94–151.5 g plant^−1^) conditions (Tables 3, 4). Under non-stress conditions, the highest SY was seen in G_58_ (185 g plant^−1^) (Table 3). However, the highest SY under drought stress was recorded in G_50_ (151 g plant^−1^) (Table 4), while the lowest value was observed in G_16_ (94 g plant^−1^) (Table 4). At the end of the two-year study period, the average SY of the arugula genotypes was higher in the second year, which could be justified by the genotype × year interaction.

**Table 3:** Mean comparisons of the different traits of the 64 arugula genotypes treated under non-stress conditions averaged over the two study years (2019 and 2020).

Genotype	SY (g plant^−1^)	Chla (mg g^−1^ FW)	Chlb (mg g^−1^ FW)	TChl (mg g^−1^ FW)	Car (mg g^−1^FW)	POX (µmol min^−1^ mg^−1^ protein)	APX (units mg^−1^ protein)	CAT (units mg^−1^ protein)	MDA (nmol g^−1^ FW)	Proline (μmolg^−1^ FW)	RWC (%)	TPC (mg TAE g^−1^ DW)	TFD (mg QE g^−1^ DW)
G1	143.25 ± 6.84	0.12 ± 0.002	0.07 ± 0.001	0.19 ± 0.003	0.33 ± 0.006	0.13 ± 0.01	5.85 ± 0.22	0.11 ± 0.001	0.1 ± 0.004	0.19 ± 0.002	78.05 ± 0.79	37.25 ± 0.66	2.71 ± 0.16
G2	159 ± 19.73	0.14 ± 0.003	0.08 ± 0.002	0.22 ± 0.004	0.35 ± 0.009	0.15 ± 0.011	5.21 ± 0.24	0.12 ± 0.002	0.08 ± 0.003	0.19 ± 0.002	83.73 ± 0.98	36.6 ± 0.5	3.51 ± 0.24
G3	158.42 ± 22.73	0.11 ± 0.004	0.07 ± 0	0.18 ± 0.004	0.4 ± 0.009	0.14 ± 0.012	5.2 ± 0.29	0.11 ± 0.001	0.08 ± 0.003	0.2 ± 0.001	88.12 ± 1.16	33.97 ± 0.89	4.11 ± 0.19
G4	160.5 ± 19.99	0.13 ± 0.003	0.08 ± 0.001	0.21 ± 0.004	0.34 ± 0.009	0.15 ± 0.011	4.11 ± 0.21	0.12 ± 0.002	0.08 ± 0.002	0.21 ± 0.001	77.54 ± 0.92	33.77 ± 0.59	3.65 ± 0.26
G5	157 ± 24.11	0.13 ± 0.003	0.08 ± 0.001	0.21 ± 0.004	0.33 ± 0.008	0.15 ± 0.011	4.94 ± 0.38	0.12 ± 0.001	0.08 ± 0.004	0.2 ± 0.004	82.96 ± 1	41.03 ± 0.36	4.43 ± 0.15
G6	126.92 ± 14.08	0.11 ± 0.003	0.07 ± 0.001	0.18 ± 0.005	0.33 ± 0.005	0.15 ± 0.011	5.08 ± 0.29	0.12 ± 0.002	0.1 ± 0.005	0.21 ± 0.001	85.14 ± 0.8	42.12 ± 0.81	5.13 ± 0.15
G7	107.5 ± 12.84	0.16 ± 0.003	0.1 ± 0.001	0.26 ± 0.004	0.39 ± 0.016	0.15 ± 0.011	4.73 ± 0.27	0.11 ± 0.002	0.08 ± 0.003	0.21 ± 0.001	79.94 ± 1.19	37.66 ± 0.55	4.78 ± 0.23
G8	150 ± 5.41	0.12 ± 0.003	0.07 ± 0	0.19 ± 0.003	0.32 ± 0.006	0.15 ± 0.012	5.9 ± 0.25	0.12 ± 0.001	0.09 ± 0.006	0.21 ± 0.002	85.82 ± 0.95	38.95 ± 0.48	4.28 ± 0.16
G9	129 ± 7.87	0.15 ± 0.004	0.1 ± 0.002	0.25 ± 0.006	0.42 ± 0.029	0.16 ± 0.011	4.26 ± 0.31	0.13 ± 0.001	0.11 ± 0.004	0.2 ± 0.002	83.7 ± 0.96	34.08 ± 0.49	5.84 ± 0.2
G10	141.5 ± 19.5	0.17 ± 0.003	0.1 ± 0.005	0.27 ± 0.006	0.45 ± 0.025	0.15 ± 0.012	5.77 ± 0.47	0.12 ± 0.001	0.09 ± 0.001	0.19 ± 0.002	88.57 ± 0.98	34.77 ± 0.51	6.26 ± 0.13
G11	121.92 ± 7.59	0.13 ± 0.003	0.08 ± 0.001	0.21 ± 0.003	0.38 ± 0.012	0.14 ± 0.011	6.36 ± 0.22	0.11 ± 0.002	0.09 ± 0.016	0.21 ± 0.001	80.18 ± 0.82	33.78 ± 0.5	5.3 ± 0.15
G12	118.42 ± 9.78	0.13 ± 0.003	0.08 ± 0.001	0.21 ± 0.005	0.34 ± 0.008	0.15 ± 0.011	4.6 ± 0.44	0.11 ± 0.001	0.08 ± 0.003	0.21 ± 0.002	90.69 ± 0.78	31.22 ± 0.49	4.89 ± 0.14
G13	184.83 ± 19.43	0.11 ± 0.003	0.07 ± 0.001	0.18 ± 0.003	0.34 ± 0.008	0.13 ± 0.01	5.21 ± 0.35	0.11 ± 0.001	0.09 ± 0.016	0.19 ± 0.004	76.94 ± 0.72	25.22 ± 3.42	3.86 ± 0.16
G14	162.33 ± 23.91	0.13 ± 0.003	0.08 ± 0.001	0.21 ± 0.004	0.35 ± 0.01	0.11 ± 0.009	5.02 ± 0.3	0.11 ± 0.002	0.08 ± 0.006	0.2 ± 0.002	79.65 ± 0.6	27.57 ± 0.56	1.98 ± 0.31
G15	162.83 ± 16.8	0.16 ± 0.003	0.09 ± 0.003	0.26 ± 0.006	0.42 ± 0.021	0.16 ± 0.011	3.79 ± 0.25	0.11 ± 0.003	0.08 ± 0.004	0.2 ± 0.002	75.84 ± 0.5	30.18 ± 0.41	3.14 ± 0.24
G16	163.33 ± 14.93	0.1 ± 0.004	0.07 ± 0.002	0.17 ± 0.005	0.33 ± 0.01	0.16 ± 0.011	5.32 ± 0.32	0.11 ± 0.002	0.07 ± 0.002	0.19 ± 0.002	72.62 ± 0.64	32.06 ± 0.49	3.94 ± 0.17
G17	164.5 ± 16.1	0.15 ± 0.004	0.09 ± 0.002	0.24 ± 0.006	0.42 ± 0.014	0.13 ± 0.01	4.88 ± 0.42	0.12 ± 0.002	0.07 ± 0.002	0.21 ± 0.002	69.7 ± 0.81	29.75 ± 0.47	2.27 ± 0.22
G18	178 ± 15.43	0.11 ± 0.003	0.07 ± 0.001	0.18 ± 0.004	0.32 ± 0.012	0.13 ± 0.009	6.06 ± 0.2	0.1 ± 0.001	0.08 ± 0.002	0.2 ± 0.003	67.15 ± 0.55	28.01 ± 0.48	3.35 ± 0.15
G19	171.67 ± 13.53	0.11 ± 0.001	0.08 ± 0.001	0.19 ± 0.002	0.33 ± 0.011	0.16 ± 0.011	4.33 ± 0.27	0.12 ± 0.002	0.09 ± 0.002	0.21 ± 0.001	65.15 ± 0.52	29.81 ± 0.35	4.04 ± 0.21
G20	129.75 ± 15.22	0.14 ± 0.019	0.1 ± 0.001	0.24 ± 0.019	0.44 ± 0.012	0.16 ± 0.011	5.72 ± 0.14	0.11 ± 0.003	0.1 ± 0.003	0.19 ± 0.002	72.83 ± 0.72	28.04 ± 0.44	4.12 ± 0.16
G21	165.42 ± 23.84	0.18 ± 0.003	0.1 ± 0.002	0.27 ± 0.004	0.49 ± 0.019	0.16 ± 0.01	3.71 ± 0.17	0.11 ± 0.002	0.09 ± 0.001	0.19 ± 0.003	72.93 ± 0.7	31.1 ± 0.41	4.75 ± 0.15
G22	170.5 ± 17.12	0.12 ± 0.004	0.07 ± 0.001	0.2 ± 0.004	0.32 ± 0.011	0.15 ± 0.011	3.4 ± 0.23	0.12 ± 0.002	0.08 ± 0.002	0.2 ± 0.001	76.06 ± 0.6	30.61 ± 0.52	3.35 ± 0.16
G23	142.42 ± 22.68	0.13 ± 0.003	0.08 ± 0.001	0.21 ± 0.004	0.35 ± 0.012	0.16 ± 0.012	4.15 ± 0.19	0.12 ± 0.005	0.07 ± 0.003	0.21 ± 0.001	68.46 ± 0.66	28.22 ± 0.7	4.35 ± 0.21
G24	171.75 ± 9.75	0.12 ± 0.003	0.07 ± 0.001	0.19 ± 0.004	0.32 ± 0.008	0.15 ± 0.01	4.11 ± 0.3	0.11 ± 0.003	0.07 ± 0.002	0.21 ± 0.001	71.68 ± 0.56	31.01 ± 0.49	3.34 ± 0.12
G25	138.33 ± 24.38	0.12 ± 0.004	0.08 ± 0.002	0.2 ± 0.005	0.32 ± 0.01	0.17 ± 0.011	4.03 ± 0.33	0.12 ± 0.001	0.07 ± 0.003	0.21 ± 0.001	75.25 ± 0.63	32.69 ± 0.37	3.48 ± 0.13
G26	145.92 ± 23.69	0.12 ± 0.007	0.08 ± 0.002	0.2 ± 0.008	0.37 ± 0.009	0.15 ± 0.011	5.27 ± 0.24	0.11 ± 0.002	0.07 ± 0.002	0.21 ± 0.002	79.06 ± 0.69	33.06 ± 0.45	4.45 ± 0.14
G27	174.08 ± 25.48	0.14 ± 0.005	0.09 ± 0.003	0.23 ± 0.008	0.39 ± 0.012	0.15 ± 0.01	5.34 ± 0.3	0.12 ± 0.002	0.05 ± 0.001	0.18 ± 0.002	81.67 ± 0.52	33.86 ± 0.46	5.09 ± 0.21
G28	136.25 ± 16	0.13 ± 0.003	0.09 ± 0.003	0.21 ± 0.006	0.35 ± 0.012	0.13 ± 0.01	3.85 ± 0.22	0.1 ± 0.001	0.1 ± 0.002	0.2 ± 0.001	85.37 ± 0.56	32.23 ± 0.53	3.71 ± 0.12
G29	160.83 ± 17.32	0.17 ± 0.004	0.09 ± 0.005	0.26 ± 0.008	0.5 ± 0.022	0.16 ± 0.012	4.19 ± 0.47	0.1 ± 0.001	0.08 ± 0.003	0.19 ± 0.003	83.45 ± 0.63	35.87 ± 0.65	4.39 ± 0.16
G30	143.67 ± 20.2	0.26 ± 0.137	0.1 ± 0.003	0.36 ± 0.138	0.34 ± 0.01	0.16 ± 0.011	5.19 ± 0.23	0.1 ± 0.001	0.08 ± 0.003	0.2 ± 0.001	79.93 ± 0.7	27.29 ± 4.77	3.82 ± 0.16
G31	138.17 ± 18.19	0.13 ± 0.004	0.08 ± 0.003	0.21 ± 0.006	0.38 ± 0.011	0.17 ± 0.011	4.1 ± 0.22	0.13 ± 0.003	0.08 ± 0.004	0.2 ± 0.002	82.45 ± 0.6	25.72 ± 3.3	4.89 ± 0.19
G32	137.5 ± 13.35	0.12 ± 0.015	0.08 ± 0.003	0.2 ± 0.016	0.37 ± 0.01	0.16 ± 0.011	5.13 ± 0.2	0.1 ± 0.002	0.08 ± 0.003	0.2 ± 0.001	78.07 ± 0.75	30.04 ± 0.42	3.38 ± 0.13
G33	155.58 ± 27.21	0.13 ± 0.003	0.08 ± 0.002	0.21 ± 0.006	0.35 ± 0.01	0.15 ± 0.011	5.78 ± 0.21	0.11 ± 0.002	0.08 ± 0.002	0.2 ± 0.002	79.95 ± 0.72	27.41 ± 0.43	4.69 ± 0.14
G34	155.83 ± 14.33	0.15 ± 0.003	0.08 ± 0.003	0.23 ± 0.006	0.41 ± 0.01	0.16 ± 0.01	4.85 ± 0.2	0.11 ± 0.004	0.09 ± 0.002	0.19 ± 0.002	83.65 ± 0.62	29.63 ± 0.42	5.32 ± 0.15
G35	162.08 ± 30.69	0.13 ± 0.003	0.08 ± 0.002	0.2 ± 0.005	0.34 ± 0.011	0.15 ± 0.01	3.32 ± 0.21	0.13 ± 0.004	0.08 ± 0.004	0.2 ± 0.002	86.09 ± 0.59	27.38 ± 0.46	5.28 ± 0.14
G36	155.83 ± 27.86	0.11 ± 0.003	0.07 ± 0.002	0.18 ± 0.004	0.34 ± 0.011	0.16 ± 0.011	4.78 ± 0.21	0.11 ± 0.003	0.11 ± 0.005	0.2 ± 0.005	76.35 ± 0.7	27.47 ± 0.62	6.23 ± 0.16
G37	130.42 ± 10.13	0.14 ± 0.003	0.08 ± 0.001	0.21 ± 0.004	0.36 ± 0.009	0.13 ± 0.011	6.48 ± 0.13	0.11 ± 0.002	0.09 ± 0.005	0.2 ± 0.002	78.64 ± 0.62	26.43 ± 0.62	3.48 ± 0.14
G38	139.83 ± 24.23	0.11 ± 0.003	0.08 ± 0.003	0.19 ± 0.006	0.35 ± 0.006	0.13 ± 0.024	5.11 ± 0.19	0.1 ± 0.001	0.09 ± 0.005	0.2 ± 0.003	75.92 ± 0.53	27.89 ± 0.46	3.33 ± 0.15
G39	160.17 ± 16.14	0.11 ± 0.003	0.08 ± 0.003	0.19 ± 0.006	0.31 ± 0.009	0.16 ± 0.01	4.19 ± 0.2	0.1 ± 0.002	0.09 ± 0.003	0.19 ± 0.002	74.15 ± 0.72	29.65 ± 0.53	4.23 ± 0.14
G40	^¥^149.83 ± 14.89	0.13 ± 0.003	0.08 ± 0.003	0.2 ± 0.007	0.33 ± 0.017	0.18 ± 0.011	5.66 ± 0.15	0.11 ± 0.005	0.08 ± 0.002	0.19 ± 0.003	72.55 ± 0.61	27.04 ± 0.49	3.41 ± 0.13
G41	146.5 ± 11.88	0.12 ± 0.004	0.08 ± 0.002	0.2 ± 0.006	0.32 ± 0.013	0.16 ± 0.011	4.78 ± 0.16	0.1 ± 0.002	0.08 ± 0.004	0.2 ± 0.003	71.34 ± 0.54	26.14 ± 0.55	3.24 ± 0.29
G42	161.42 ± 29.69	0.14 ± 0.003	0.08 ± 0.002	0.22 ± 0.005	0.37 ± 0.01	0.17 ± 0.01	4.09 ± 0.19	0.11 ± 0.003	0.09 ± 0.004	0.19 ± 0.002	69.21 ± 0.82	26.23 ± 0.54	5.42 ± 0.18
G43	141.5 ± 20.42	0.13 ± 0.004	0.09 ± 0.003	0.21 ± 0.007	0.34 ± 0.014	0.17 ± 0.01	5.68 ± 0.16	0.11 ± 0.003	0.08 ± 0.004	0.19 ± 0.002	67.97 ± 0.73	24.58 ± 0.58	2.84 ± 0.17
G44	147.5 ± 24.4	0.15 ± 0.004	0.08 ± 0.002	0.23 ± 0.006	0.4 ± 0.013	0.17 ± 0.011	5.83 ± 0.2	0.1 ± 0.001	0.11 ± 0.004	0.2 ± 0.001	72.02 ± 0.72	20.98 ± 2.93	3.9 ± 0.16
G45	167 ± 21.36	0.12 ± 0.003	0.09 ± 0.004	0.2 ± 0.003	0.35 ± 0.012	0.24 ± 0.042	4.15 ± 0.18	0.11 ± 0.003	0.08 ± 0.001	0.21 ± 0.002	68.12 ± 0.71	23.02 ± 0.54	2.27 ± 0.17
G46	149.5 ± 20.44	0.15 ± 0.003	0.09 ± 0.004	0.24 ± 0.007	0.42 ± 0.014	0.18 ± 0.01	3.27 ± 0.22	0.11 ± 0.003	0.08 ± 0.004	0.19 ± 0.002	71.32 ± 0.56	22.55 ± 0.43	3.29 ± 0.13
G47	171.42 ± 27.57	0.13 ± 0.002	0.08 ± 0.003	0.21 ± 0.005	0.36 ± 0.005	0.18 ± 0.011	4.19 ± 0.2	0.13 ± 0.006	0.09 ± 0.004	0.2 ± 0.002	70.05 ± 0.72	21.39 ± 2.97	2.7 ± 0.18
G48	171.33 ± 16.78	0.12 ± 0.003	0.08 ± 0.003	0.2 ± 0.006	0.32 ± 0.009	0.18 ± 0.011	5.14 ± 0.2	0.12 ± 0.002	0.08 ± 0.003	0.21 ± 0.003	67.31 ± 0.65	22.75 ± 0.63	2.69 ± 0.11
G49	169.33 ± 20.68	0.11 ± 0.004	0.07 ± 0.003	0.19 ± 0.006	0.31 ± 0.006	0.18 ± 0.01	6.06 ± 0.2	0.11 ± 0.005	0.1 ± 0.003	0.2 ± 0.003	74.74 ± 0.64	23.88 ± 0.52	2.61 ± 0.18
G50	157.33 ± 17.96	0.13 ± 0.003	0.08 ± 0.002	0.21 ± 0.006	0.35 ± 0.013	0.18 ± 0.011	4.7 ± 0.16	0.11 ± 0.003	0.08 ± 0.004	0.19 ± 0.002	78.86 ± 0.7	26.21 ± 0.36	3.33 ± 0.19
G51	153.42 ± 22.73	0.13 ± 0.006	0.08 ± 0.004	0.21 ± 0.01	0.36 ± 0.014	0.18 ± 0.01	4.16 ± 0.19	0.1 ± 0.003	0.08 ± 0.004	0.2 ± 0.003	81.72 ± 0.66	27.47 ± 0.43	4.36 ± 0.24
G52	184.92 ± 32.59	0.12 ± 0.003	0.08 ± 0.003	0.2 ± 0.006	0.31 ± 0.011	0.15 ± 0.011	5.66 ± 0.14	0.11 ± 0.004	0.08 ± 0.001	0.2 ± 0.002	85.04 ± 0.48	28.26 ± 0.49	3.91 ± 0.27
G53	145.5 ± 17.64	0.11 ± 0.004	0.08 ± 0.003	0.19 ± 0.006	0.31 ± 0.01	0.12 ± 0.011	4.19 ± 0.21	0.12 ± 0.006	0.09 ± 0.002	0.2 ± 0.003	79.73 ± 0.61	28.45 ± 0.33	4 ± 0.24
G54	157.83 ± 28.46	0.12 ± 0.003	0.08 ± 0.003	0.2 ± 0.006	0.31 ± 0.013	0.16 ± 0.011	4.84 ± 0.2	0.1 ± 0.019	0.08 ± 0.004	0.19 ± 0.003	77.2 ± 0.57	29.03 ± 0.46	2.67 ± 0.19
G55	174 ± 28.72	0.12 ± 0.003	0.08 ± 0.003	0.2 ± 0.005	0.33 ± 0.009	0.14 ± 0.011	4.13 ± 0.19	0.12 ± 0.004	0.07 ± 0.003	0.19 ± 0.002	82.55 ± 0.58	30.49 ± 0.34	3.24 ± 0.18
G56	184.5 ± 17.23	0.15 ± 0.004	0.08 ± 0.004	0.23 ± 0.008	0.41 ± 0.016	0.17 ± 0.01	4.11 ± 0.21	0.13 ± 0.003	0.09 ± 0.005	0.19 ± 0.001	80.03 ± 0.68	27.09 ± 0.49	3.28 ± 0.14
G57	167.5 ± 24.02	0.11 ± 0.004	0.07 ± 0.002	0.18 ± 0.006	0.3 ± 0.006	0.17 ± 0.011	5.06 ± 0.22	0.1 ± 0.001	0.07 ± 0.008	0.19 ± 0.002	76.72 ± 0.59	27.53 ± 0.65	3.55 ± 0.11
G58	185.17 ± 21.58	0.13 ± 0.003	0.08 ± 0.002	0.2 ± 0.005	0.33 ± 0.01	0.18 ± 0.011	3.2 ± 0.2	0.11 ± 0.003	0.09 ± 0.004	0.19 ± 0.002	81.83 ± 0.66	28.31 ± 0.74	3.91 ± 0.19
G59	181.33 ± 27.17	0.12 ± 0.004	0.07 ± 0.002	0.19 ± 0.007	0.33 ± 0.011	0.17 ± 0.011	6.07 ± 0.21	0.1 ± 0.003	0.09 ± 0.002	0.19 ± 0.003	76.98 ± 0.48	26.86 ± 0.47	3.6 ± 0.12
G60	166.83 ± 23.9	0.17 ± 0.004	0.1 ± 0.002	0.27 ± 0.005	0.42 ± 0.018	0.17 ± 0.01	4.8 ± 0.18	0.11 ± 0.003	0.09 ± 0.003	0.2 ± 0.003	82.65 ± 0.64	27.26 ± 0.71	4.44 ± 0.21
G61	163.67 ± 22.68	0.15 ± 0.003	0.08 ± 0.002	0.23 ± 0.005	0.39 ± 0.013	0.18 ± 0.011	4.07 ± 0.2	0.12 ± 0.002	0.07 ± 0.006	0.19 ± 0.003	81.41 ± 0.54	28.39 ± 0.35	4.24 ± 0.15
G62	166.5 ± 23.21	0.15 ± 0.005	0.09 ± 0.003	0.24 ± 0.007	0.41 ± 0.014	0.17 ± 0.011	3.19 ± 0.2	0.11 ± 0.004	0.08 ± 0.003	0.19 ± 0.002	79.1 ± 0.7	28.93 ± 0.46	3.38 ± 0.15
G63	166.83 ± 21.91	0.15 ± 0.005	0.1 ± 0.002	0.25 ± 0.007	0.39 ± 0.007	0.17 ± 0.01	4.64 ± 0.15	0.1 ± 0.001	0.09 ± 0.005	0.19 ± 0.002	78.28 ± 0.61	28.06 ± 0.42	4.38 ± 0.16
G64	145.83 ± 14.14	0.12 ± 0.003	0.08 ± 0.002	0.2 ± 0.005	0.36 ± 0.01	0.18 ± 0.012	4.14 ± 0.16	0.1 ± 0.002	0.07 ± 0.005	0.2 ± 0.003	82.16 ± 0.5	28.6 ± 0.64	4.77 ± 0.17
**LSD (5%)**	6.9	0.005	0.004	0.052	0.024	0.029	0.96	0.006	0.019	0.005	1.47	1.35	0.68

*SY* seed yield per plant, *Chl a* chlorophyll a concentration, *Chl b* chlorophyllb concentrations, *TChl* total chlorophyll, *Car* carotenoids, *POX* peroxidase, *APX* ascorbate peroxidase, *CAT* catalase, *MDA* malondialdehyde, *RWC* relative water content, *TPC* total phenolics content, *TFD* total flavonoids.

^¥^The data are presented as mean ± standard error (SE).

**Table 4:** Mean comparisons of the different traits of the 64 arugula genotypes treated under stress conditions averaged over the two study years (2019 and 2020).

Genotype	SY (g plant^−1^)	Chl a (mg g^−1^ FW)	Chl b (mg g^−1^ FW)	TChl (mg g^−1^ FW)	Car (mg g^−1^ FW)	POX (µmol min^−1^ mg^−1^ protein)	APX (units mg^−1^ protein )	CAT (units mg^−1^ protein^−1^)	MDA (nmol g^−1^ FW)	Proline (μmolg^−1^ FW)	RWC (%)	TPC (mg TAE g^−1^ DW)	TFD (mg QE g^−1^ DW)
G1	128.2 ± 9.59	0.1 ± 0.001	0.05 ± 0.001	0.15 ± 0.001	0.27 ± 0.006	0.21 ± 0.01	7.74 ± 0.23	0.19 ± 0.001	0.17 ± 0.003	0.24 ± 0.002	66.8 ± 0.96	46.83 ± 0.73	3.62 ± 0.15
G2	105.8 ± 19.15	0.09 ± 0.003	0.05 ± 0.001	0.14 ± 0.003	0.25 ± 0.016	0.22 ± 0.01	6.54 ± 0.17	0.2 ± 0.003	0.17 ± 0.007	0.25 ± 0.004	69 ± 1.26	44.21 ± 0.58	4.35 ± 0.27
G3	99.7 ± 26.27	0.09 ± 0.002	0.05 ± 0.002	0.14 ± 0.002	0.26 ± 0.011	0.23 ± 0.01	7.21 ± 0.3	0.2 ± 0	0.18 ± 0.005	0.25 ± 0.003	70.27 ± 1.18	51.23 ± 0.58	5.36 ± 0.2
G4	107.8 ± 17.47	0.11 ± 0.002	0.06 ± 0	0.17 ± 0.002	0.26 ± 0.009	0.22 ± 0.011	7.55 ± 0.21	0.19 ± 0.002	0.17 ± 0.005	0.27 ± 0.003	69.24 ± 1.05	48.07 ± 0.6	5.31 ± 0.29
G5	103.1 ± 22.35	0.12 ± 0.002	0.07 ± 0.001	0.19 ± 0.003	0.33 ± 0.006	0.23 ± 0.01	7.18 ± 0.35	0.21 ± 0.002	0.17 ± 0.006	0.24 ± 0.005	72.54 ± 1.15	44.13 ± 4.36	6.56 ± 0.2
G6	116 ± 15.09	0.1 ± 0.002	0.05 ± 0.001	0.15 ± 0.002	0.28 ± 0.008	0.22 ± 0.011	7.63 ± 0.27	0.19 ± 0.001	0.2 ± 0.005	0.25 ± 0.004	71.95 ± 0.98	41.73 ± 0.65	5.67 ± 0.17
G7	105.3 ± 13.12	0.09 ± 0.002	0.05 ± 0.001	0.14 ± 0.004	0.25 ± 0.009	0.19 ± 0.01	7.18 ± 0.27	0.19 ± 0.001	0.18 ± 0.005	0.24 ± 0.002	63.38 ± 0.97	37.92 ± 0.56	3.67 ± 0.23
G8	124.3 ± 12.56	0.1 ± 0.002	0.06 ± 0.001	0.15 ± 0.003	0.25 ± 0.01	0.24 ± 0.01	6.7 ± 0.24	0.19 ± 0.002	0.17 ± 0.004	0.24 ± 0.002	58.87 ± 1.18	41.47 ± 0.51	4.11 ± 0.19
G9	124.3 ± 8.88	0.1 ± 0.005	0.05 ± 0.002	0.15 ± 0.006	0.28 ± 0.017	0.2 ± 0.009	7.38 ± 0.27	0.19 ± 0.002	0.15 ± 0.007	0.25 ± 0.003	53.36 ± 1.03	39.13 ± 0.69	3.22 ± 0.16
G10	108.9 ± 14.85	0.11 ± 0.004	0.06 ± 0.003	0.17 ± 0.006	0.27 ± 0.015	0.23 ± 0.01	7.31 ± 0.47	0.18 ± 0.002	0.17 ± 0.004	0.24 ± 0.004	53.55 ± 1.08	39.2 ± 0.53	4.73 ± 0.19
G11	115.3 ± 7.15	0.11 ± 0.003	0.05 ± 0.001	0.17 ± 0.003	0.32 ± 0.01	0.23 ± 0.01	5.83 ± 0.24	0.19 ± 0.003	0.16 ± 0.003	0.23 ± 0.006	59.35 ± 1.05	41.24 ± 0.7	4.65 ± 0.18
G12	112.5 ± 7.28	0.09 ± 0.002	0.05 ± 0.001	0.14 ± 0.003	0.25 ± 0.005	0.23 ± 0.009	6.32 ± 0.46	0.19 ± 0.002	0.18 ± 0.002	0.25 ± 0.005	54.89 ± 0.99	39.89 ± 0.48	4.29 ± 0.15
G13	105 ± 14.29	0.08 ± 0.002	0.05 ± 0.001	0.13 ± 0.002	0.25 ± 0.004	0.24 ± 0.01	6.68 ± 0.31	0.19 ± 0.003	0.17 ± 0.005	0.25 ± 0.004	61.68 ± 1.06	43.42 ± 0.6	4.41 ± 0.16
G14	99.3 ± 27.57	0.1 ± 0.002	0.05 ± 0.082	0.16 ± 0.083	0.28 ± 0.013	0.21 ± 0.01	6.74 ± 0.3	0.19 ± 0.003	0.16 ± 0.003	0.23 ± 0.003	68.44 ± 0.75	43.5 ± 0.57	5.18 ± 0.18
G15	115 ± 15.8	0.12 ± 0.005	0.06 ± 0.002	0.18 ± 0.007	0.29 ± 0.014	0.24 ± 0.01	6.75 ± 0.26	0.18 ± 0.005	0.15 ± 0.002	0.24 ± 0.004	66.32 ± 0.72	44.32 ± 0.49	4.65 ± 0.22
G16	94 ± 12.19	0.11 ± 0.003	0.05 ± 0.001	0.15 ± 0.004	0.28 ± 0.005	0.24 ± 0.009	6.5 ± 0.31	0.19 ± 0.003	0.16 ± 0.005	0.24 ± 0.003	64.72 ± 0.77	40.41 ± 0.59	4.72 ± 0.18
G17	103.7 ± 21.71	0.1 ± 0.003	0.05 ± 0.002	0.15 ± 0.004	0.3 ± 0.007	0.23 ± 0.01	7.25 ± 0.41	0.18 ± 0.002	0.17 ± 0.007	0.23 ± 0.004	66.02 ± 0.69	39 ± 0.48	5.77 ± 0.22
G18	97.1 ± 16.79	0.09 ± 0.001	0.05 ± 0.001	0.13 ± 0.002	0.26 ± 0.007	0.23 ± 0.01	6.24 ± 0.2	0.19 ± 0.001	0.2 ± 0.005	0.24 ± 0.004	65.67 ± 0.68	37.97 ± 0.6	6.35 ± 0.18
G19	103.5 ± 16.16	0.09 ± 0.001	0.05 ± 0.002	0.14 ± 0.002	0.27 ± 0.01	0.22 ± 0.009	7.94 ± 0.22	0.18 ± 0.004	0.18 ± 0.006	0.24 ± 0.003	62.04 ± 0.69	37.86 ± 0.56	3.86 ± 0.18
G20	124 ± 13.16	0.09 ± 0.007	0.04 ± 0.002	0.13 ± 0.005	0.24 ± 0.01	0.24 ± 0.01	7.28 ± 0.15	0.19 ± 0.003	0.16 ± 0.005	0.23 ± 0.004	58.38 ± 0.83	38.72 ± 0.54	4.34 ± 0.22
G21	119.7 ± 4.07	0.1 ± 0.001	0.05 ± 0.003	0.15 ± 0.003	0.27 ± 0.01	0.24 ± 0.009	6.41 ± 0.16	0.18 ± 0.003	0.18 ± 0.002	0.23 ± 0.005	54.62 ± 0.69	36.36 ± 0.68	4.94 ± 0.15
G22	100.2 ± 20.84	0.1 ± 0.002	0.05 ± 0.001	0.15 ± 0.003	0.3 ± 0.006	0.25 ± 0.012	7.54 ± 0.16	0.18 ± 0.002	0.18 ± 0.005	0.24 ± 0.004	54.68 ± 0.71	34.98 ± 0.55	4.02 ± 0.2
G23	100.9 ± 20.27	0.1 ± 0.003	0.05 ± 0.002	0.16 ± 0.005	0.28 ± 0.01	0.25 ± 0.009	5.9 ± 0.13	0.18 ± 0.006	0.17 ± 0.003	0.24 ± 0.004	54.2 ± 0.73	33.15 ± 0.63	3.75 ± 0.24
G24	121.7 ± 19.35	0.09 ± 0.003	0.05 ± 0.002	0.14 ± 0.004	0.26 ± 0.003	0.26 ± 0.011	6.73 ± 0.26	0.19 ± 0.005	0.14 ± 0.005	0.24 ± 0.003	53.02 ± 0.72	34.22 ± 0.58	3.25 ± 0.22
G25	113.9 ± 17.64	0.09 ± 0.004	0.05 ± 0.002	0.14 ± 0.005	0.24 ± 0.01	0.29 ± 0.008	7.21 ± 0.31	0.18 ± 0.002	0.18 ± 0.007	0.23 ± 0.003	61.73 ± 0.77	35.83 ± 0.49	3.4 ± 0.22
G26	119.2 ± 19.34	0.09 ± 0.006	0.05 ± 0.002	0.14 ± 0.008	0.25 ± 0.018	0.23 ± 0.01	6.95 ± 0.2	0.18 ± 0.002	0.16 ± 0.004	0.24 ± 0.003	67.94 ± 0.84	38.58 ± 0.55	4.77 ± 0.19
G27	103.7 ± 22.07	0.08 ± 0.003	0.05 ± 0.004	0.13 ± 0.007	0.25 ± 0.019	0.21 ± 0.016	6.55 ± 0.25	0.19 ± 0.002	0.19 ± 0.004	0.23 ± 0.005	62.87 ± 0.69	39.16 ± 0.56	3.9 ± 0.14
G28	126.4 ± 20.7	0.1 ± 0.001	0.05 ± 0.003	0.15 ± 0.004	0.28 ± 0.006	0.23 ± 0.013	6.2 ± 0.24	0.2 ± 0.003	0.18 ± 0.005	0.23 ± 0.003	65.82 ± 0.67	39.34 ± 0.59	3.7 ± 0.13
G29	126.3 ± 2.69	0.08 ± 0.002	0.04 ± 0.005	0.13 ± 0.007	0.23 ± 0.023	0.25 ± 0.01	6.27 ± 0.48	0.18 ± 0.003	0.17 ± 0.006	0.23 ± 0.005	63.88 ± 0.77	38.49 ± 0.63	4.45 ± 0.16
G30	127.5 ± 16.4	0.11 ± 0.001	0.06 ± 0.003	0.17 ± 0.004	0.29 ± 0.009	0.24 ± 0.009	7.27 ± 0.31	0.18 ± 0.003	0.17 ± 0.005	0.24 ± 0.004	64.67 ± 0.66	37.69 ± 0.55	4.67 ± 0.18
G31	128.1 ± 15.67	0.11 ± 0.003	0.06 ± 0.003	0.17 ± 0.004	0.32 ± 0.019	0.25 ± 0.01	5.68 ± 0.22	0.19 ± 0.003	0.17 ± 0.005	0.24 ± 0.003	64.75 ± 0.77	39.42 ± 0.51	4.26 ± 0.16
G32	121.5 ± 15.36	0.11 ± 0.001	0.06 ± 0.003	0.16 ± 0.003	0.31 ± 0.007	0.25 ± 0.01	6.65 ± 0.21	0.17 ± 0.003	0.17 ± 0.004	0.22 ± 0.003	64.9 ± 0.81	39.05 ± 0.55	5.05 ± 0.18
G33	136.8 ± 16.27	0.1 ± 0.001	0.05 ± 0.003	0.16 ± 0.004	0.31 ± 0.007	0.26 ± 0.01	8.14 ± 0.31	0.2 ± 0.003	0.19 ± 0.004	0.25 ± 0.005	71.17 ± 0.89	49.09 ± 0.57	4.24 ± 0.21
G34	143.3 ± 18.28	0.1 ± 0.003	0.05 ± 0.003	0.15 ± 0.007	0.28 ± 0.009	0.27 ± 0.011	7.21 ± 0.21	0.2 ± 0.004	0.19 ± 0.006	0.26 ± 0.005	73.13 ± 0.77	46.55 ± 0.41	5.04 ± 0.15
G35	136.1 ± 32.13	0.1 ± 0.002	0.05 ± 0.005	0.15 ± 0.004	0.28 ± 0.01	0.27 ± 0.01	7.45 ± 0.18	0.2 ± 0.004	0.2 ± 0.006	0.26 ± 0.004	74.37 ± 0.74	49.47 ± 0.62	5.96 ± 0.18
G36	133.2 ± 31.1	0.12 ± 0.001	0.06 ± 0.001	0.18 ± 0.001	0.3 ± 0.006	0.27 ± 0.009	7.88 ± 0.21	0.2 ± 0.003	0.19 ± 0.004	0.27 ± 0.004	73.43 ± 0.73	50.14 ± 0.54	5.82 ± 0.19
G37	128.8 ± 7.8	0.14 ± 0.002	0.07 ± 0.002	0.21 ± 0.004	0.4 ± 0.009	0.27 ± 0.01	7.64 ± 0.2	0.22 ± 0.003	0.2 ± 0.002	0.25 ± 0.005	76.57 ± 0.73	46.5 ± 0.52	7.19 ± 0.21
G38	128.5 ± 25.02	0.1 ± 0.002	0.06 ± 0.002	0.16 ± 0.002	0.3 ± 0.007	0.26 ± 0.01	7.95 ± 0.12	0.2 ± 0.004	0.21 ± 0.003	0.27 ± 0.005	76.35 ± 0.71	43.97 ± 0.53	6.21 ± 0.17
G39	141.1 ± 20.35	0.1 ± 0.002	0.08 ± 0.001	0.18 ± 0.004	0.28 ± 0.008	0.24 ± 0.01	7.66 ± 0.21	0.2 ± 0.002	0.2 ± 0.006	0.25 ± 0.004	67.15 ± 0.57	40.05 ± 0.48	4.2 ± 0.18
G40	139.9 ± 14.33	0.11 ± 0.002	0.06 ± 0.003	0.18 ± 0.004	0.28 ± 0.008	0.28 ± 0.011	7.06 ± 0.15	0.2 ± 0.003	0.19 ± 0.004	0.25 ± 0.005	61.92 ± 0.77	43.42 ± 0.55	3.77 ± 0.3
G41	144.3 ± 17.45	0.1 ± 0.003	0.06 ± 0.002	0.16 ± 0.004	0.3 ± 0.005	0.25 ± 0.01	8.03 ± 0.18	0.2 ± 0.003	0.18 ± 0.004	0.26 ± 0.005	56.37 ± 0.96	41.26 ± 0.55	5.26 ± 0.19
G42	134.4 ± 23.78	0.1 ± 0.002	0.07 ± 0.001	0.16 ± 0.003	0.31 ± 0.012	0.28 ± 0.01	7.81 ± 0.19	0.2 ± 0.003	0.19 ± 0.002	0.26 ± 0.004	56.56 ± 0.81	41.44 ± 0.58	5.21 ± 0.16
G43	134.8 ± 20.17	0.12 ± 0.004	0.06 ± 0.003	0.18 ± 0.007	0.33 ± 0.008	0.27 ± 0.01	6.18 ± 0.31	0.2 ± 0.004	0.17 ± 0.003	0.25 ± 0.005	62.42 ± 0.72	43.65 ± 0.59	5.11 ± 0.17
G44	140 ± 24.99	0.1 ± 0.003	0.05 ± 0.002	0.16 ± 0.004	0.27 ± 0.008	0.27 ± 0.01	6.71 ± 0.3	0.21 ± 0.004	0.2 ± 0.004	0.27 ± 0.004	57.75 ± 0.82	41.91 ± 0.52	5.16 ± 0.4
G45	138.6 ± 25.72	0.1 ± 0.004	0.06 ± 0.004	0.16 ± 0.002	0.31 ± 0.006	0.27 ± 0.01	6.99 ± 0.1	0.2 ± 0.006	0.19 ± 0.003	0.27 ± 0.005	64.94 ± 0.71	45.38 ± 0.59	5.63 ± 0.18
G46	143 ± 19.97	0.11 ± 0.002	0.07 ± 0.004	0.18 ± 0.006	0.33 ± 0.014	0.27 ± 0.009	7.24 ± 0.09	0.2 ± 0.002	0.18 ± 0.005	0.25 ± 0.003	71.45 ± 0.82	45.54 ± 0.49	5.24 ± 0.24
G47	131.5 ± 18.72	0.13 ± 0.002	0.08 ± 0.003	0.2 ± 0.005	0.36 ± 0.008	0.28 ± 0.01	7.57 ± 0.22	0.19 ± 0.009	0.18 ± 0.027	0.25 ± 0.003	69.19 ± 0.88	46.65 ± 0.55	5.41 ± 0.12
G48	130.3 ± 20.41	0.11 ± 0.002	0.06 ± 0.003	0.17 ± 0.004	0.33 ± 0.009	0.28 ± 0.01	7.16 ± 0.27	0.2 ± 0.002	0.18 ± 0.005	0.25 ± 0.005	67.75 ± 0.79	42.29 ± 0.53	6.37 ± 0.32
G49	135.8 ± 28.48	0.11 ± 0.002	0.06 ± 0.004	0.17 ± 0.005	0.33 ± 0.008	0.28 ± 0.01	8.03 ± 0.33	0.2 ± 0.006	0.19 ± 0.006	0.25 ± 0.005	69.37 ± 9.08	40.85 ± 0.61	6.99 ± 0.22
G50	151.5 ± 19.71	0.09 ± 0.002	0.04 ± 0.003	0.12 ± 0.004	0.28 ± 0.009	0.28 ± 0.036	6.85 ± 0.18	0.21 ± 0.004	0.22 ± 0.006	0.26 ± 0.003	68.87 ± 0.74	39.84 ± 0.49	4.54 ± 0.27
G51	135.5 ± 18.43	0.1 ± 0.003	0.05 ± 0.004	0.15 ± 0.006	0.29 ± 0.011	0.26 ± 0.011	8.14 ± 0.2	0.2 ± 0.005	0.18 ± 0.004	0.26 ± 0.005	65.16 ± 0.87	39.54 ± 0.63	4.97 ± 0.27
G52	142 ± 18.14	0.09 ± 0.002	0.05 ± 0.003	0.15 ± 0.005	0.27 ± 0.01	0.29 ± 0.01	7.7 ± 0.28	0.2 ± 0.006	0.18 ± 0.005	0.25 ± 0.004	60.96 ± 0.74	40.66 ± 0.5	5.58 ± 0.25
G53	143.3 ± 13.55	0.11 ± 0.003	0.06 ± 0.003	0.16 ± 0.005	0.3 ± 0.007	0.28 ± 0.011	7.04 ± 0.23	0.2 ± 0.006	0.19 ± 0.005	0.25 ± 0.004	58.33 ± 0.76	38.38 ± 0.5	4.41 ± 0.27
G54	148.9 ± 16.86	0.11 ± 0.001	0.07 ± 0.005	0.18 ± 0.006	0.32 ± 0.006	0.29 ± 0.01	8.46 ± 0.3	0.19 ± 0.004	0.19 ± 0.003	0.25 ± 0.005	57.99 ± 0.89	36.94 ± 0.56	4 ± 0.2
G55	148.9 ± 22.94	0.1 ± 0.002	0.07 ± 0.003	0.17 ± 0.005	0.31 ± 0.008	0.29 ± 0.011	6.21 ± 0.15	0.2 ± 0.004	0.19 ± 0.003	0.26 ± 0.003	57.42 ± 0.96	35.15 ± 0.59	3.79 ± 0.21
G56	142.3 ± 16.39	0.1 ± 0.002	0.06 ± 0.004	0.16 ± 0.006	0.3 ± 0.021	0.3 ± 0.009	7.55 ± 0.28	0.21 ± 0.002	0.18 ± 0.004	0.25 ± 0.003	56.49 ± 0.72	35.88 ± 0.6	4.3 ± 0.12
G57	149.5 ± 8	0.1 ± 0.001	0.06 ± 0.002	0.15 ± 0.003	0.27 ± 0.007	0.3 ± 0.01	8.02 ± 0.23	0.2 ± 0.002	0.21 ± 0.008	0.25 ± 0.003	55.44 ± 0.72	37.77 ± 0.54	5.33 ± 0.21
G58	142 ± 7.21	0.1 ± 0.004	0.06 ± 0.003	0.16 ± 0.007	0.29 ± 0.007	0.28 ± 0.011	7.69 ± 0.22	0.2 ± 0.003	0.18 ± 0.002	0.28 ± 0.006	71.39 ± 0.88	40.88 ± 0.7	4.46 ± 0.12
G59	134.9 ± 9.97	0.09 ± 0.004	0.06 ± 0.002	0.15 ± 0.006	0.25 ± 0.009	0.26 ± 0.01	7.33 ± 0.34	0.21 ± 0.004	0.2 ± 0.003	0.25 ± 0.003	66.29 ± 0.72	41.28 ± 0.53	4.45 ± 0.22
G60	148.9 ± 18.78	0.11 ± 0.003	0.06 ± 0.002	0.17 ± 0.004	0.33 ± 0.008	0.27 ± 0.01	6.92 ± 0.19	0.21 ± 0.002	0.19 ± 0.004	0.25 ± 0.003	69.31 ± 0.72	41.84 ± 0.73	4.79 ± 0.2
G61	133.4 ± 14.3	0.09 ± 0.002	0.06 ± 0.002	0.15 ± 0.004	0.26 ± 0.006	0.29 ± 0.01	6.87 ± 0.19	0.19 ± 0.003	0.19 ± 0.008	0.25 ± 0.004	67.23 ± 0.73	40.89 ± 0.52	5.15 ± 0.21
G62	143 ± 19.55	0.12 ± 0.003	0.06 ± 0.004	0.19 ± 0.006	0.32 ± 0.01	0.29 ± 0.01	8.07 ± 0.2	0.2 ± 0.002	0.18 ± 0.004	0.25 ± 0.002	67.78 ± 0.79	39.97 ± 0.56	4.93 ± 0.18
G63	143.3 ± 18.27	0.12 ± 0.002	0.07 ± 0.002	0.19 ± 0.002	0.35 ± 0.006	0.29 ± 0.01	6.33 ± 0.2	0.2 ± 0.004	0.2 ± 0.004	0.25 ± 0.005	67.97 ± 0.69	41.62 ± 0.63	5.61 ± 0.2
G64	135.3 ± 9.14	0.1 ± 0.002	0.07 ± 0.002	0.17 ± 0.001	0.33 ± 0.008	0.3 ± 0.009	7.03 ± 0.11	0.19 ± 0.003	0.18 ± 0.003	0.26 ± 0.005	67.85 ± 0.7	41.33 ± 0.56	5.53 ± 0.2
LSD (5%)	6.9	0.005	0.004	0.052	0.024	0.029	0.96	0.006	0.019	0.005	1.47	1.35	0.68

*SY* seed yield per plant, *Chl a* chlorophyll a concentration, *Chl b* chlorophyllb concentrations, *TChl* total chlorophyll concentrations, *Car* carotenoids concentration, *POX* peroxidase, *APX* ascorbate peroxidase, *CAT* catalase, *MDA* malondialdehyde, *H*_*2*_*O*_*2*_ hydrogen peroxide, *RWC* relative water content, *TPC* total phenolic content, *TFD* total flavonoids. The data are presented as mean ± standard error (SE).

### Relative water content

Water deficiency induced a significant decrease in RWC over the two years of study (Table 2). Under non-stress conditions, the G_12_ genotype had the highest amount of RWC (90.69%) (Table 3). Under drought stress conditions, the highest RWC values were seen in the G_37_ (76.57%) and G_38_ (76.35%) genotypes, while the lowest values were observed in the G_10_ (53.55%), G_1_ (53.36%), and G_24_ (53.02%) genotypes (Table 4).

### Proline

In the two years of study, significant increases were observed under drought stress conditions in the proline concentrations of the studied genotypes (Table 2). Under non-stress conditions, the highest and lowest mean proline content values (0.22 μmolg^−1^ FW and 0.18 μmolg^−1^ FW) were recorded in the G_48_ and G_27_ genotypes, respectively (Table 3). However, under drought-stress conditions, the G_58_ and G_32_ genotypes had the highest and lowest mean proline contents of 0.28 μmolg^−1^ FW and 0.22 μmolg^−1^ FW, respectively (Table 4).

### Photosynthetic pigments

The mean comparisons revealed a significant decrease in Chla, Chlb, TChl, and carotenoid contents under drought stress during the two-year study period (Table 2). Under non-stress conditions, the highest content values for both TChl (0.36 mg g^−1^ FW) and Chla (0.27 mg g^−1^ FW), Chlb (0.11 mg g^−1^ FW) and carotenoids (0.51 mg g^−1^ FW) were observed in G_30_, G_10_ and G_29_, respectively (Table 3). On the other hand, the highest content values of TChl (0.21 mg g^−1^ FW), Chla (0.14 mg g^−1^ FW), Chlb (0.08 mg g^−1^ FW) and carotenoids (0.40 mg g^−1^ FW) under drought stress were observed in the G_37_, G_39_ and G_37_ genotypes, respectively. The lowest content values of TChl (0.12 mg g^−1^ FW) and carotenoids (0.24 mg g^−1^ FW) were seen in G_50_ and G_25,_ respectively (Table 4).

### Enzymatic antioxidants

During the two-year study period, the APX, POX, and CAT activities of the drought-stressed samples increased significantly (Table 2). The APX activity values ranged from 3.19 units mg^−1^ protein in G_62_ to 6.48 unit mg^−1^ protein in G_37_ under non-stress conditions (Table 3). Under drought stress conditions, the highest APX activity values were recorded in G_54_ (8.46 units mg^−1^ protein) (Table 4), and the lowest APX values were observed in G_23_ (5.90 units mg^−1^ protein) and G_11_ (5.834 units mg^−1^ protein) (Table 3). Under non-stress conditions, the CAT activity values ranged from 0.09 units mg^−1^ protein in G_64_ to 0.13 units mg^−1^ protein in G_9_ (Table 3). However, these values varied from 0.17 units mg^−1^ protein in G_32_ to 0.22 units mg^−1^ protein in G_37_ under drought stress conditions (Table 4). The POX activity values ranged from 0.11 µmol min^−1^ mg^−1^ protein in G_14_ to 0.24 µmol min^−1^ mg^−1^ protein in G_45_ under non-stress conditions (Table 3). Under drought stress conditions, these values varied between 0.19 µmol min^−1^ mg^−1^ protein in G_7_ and 0.30 µmol min^−1^ mg^−1^ protein in G_56_ (Table 4). The MDA concentration of the drought-stressed samples increased significantly over the study period (Table 2). The lowest MDA concentration (0.05 nmol g^−1^ FW) was seen in the G_27_ genotype under non-stress conditions (Table 3). Under drought stress conditions, the highest (0.21 nmol g^−1^ FW) and lowest (0.13 nmol g^−1^ FW) accumulations of MDA were observed in G_50_ and G_24_, respectively (Table 4).

### Total phenolic and flavonoid contents

When compared to the non-stress conditions (Table 2), a non-significant increase was observed in the TPC and TFD values of the drought-stressed samples (Table 3). Under non-stress conditions, TPC varied from 20.98 mg TAE g^−1^ DW in G_44_ to 42.12 mg TAE g^−1^ DW in G_6_ (Table 3). The TPC values of the drought-stressed samples ranged between 33.15 mg TAE g^−1^ DW in G_23_ and 51.23 mg TAE g^−1^ DW in G_3_ (Table 4). Under non-stress conditions, the TFD values varied from 1.98 mg QE g^−1^ DW in G_14_ to 6.26 and 6.23 mg QE g^−1^ DW in G_10_ and G_36_, respectively (Table 3). The TFD values ranged from 3.22 and 3.25 mg QE g^−1^ DW in G_9_ and G_24_, respectively, to 7.19 mg QE g^−1^ DW in G_37_ under drought stress conditions (Table 4).

### Correlation analysis

**Figure 1:**
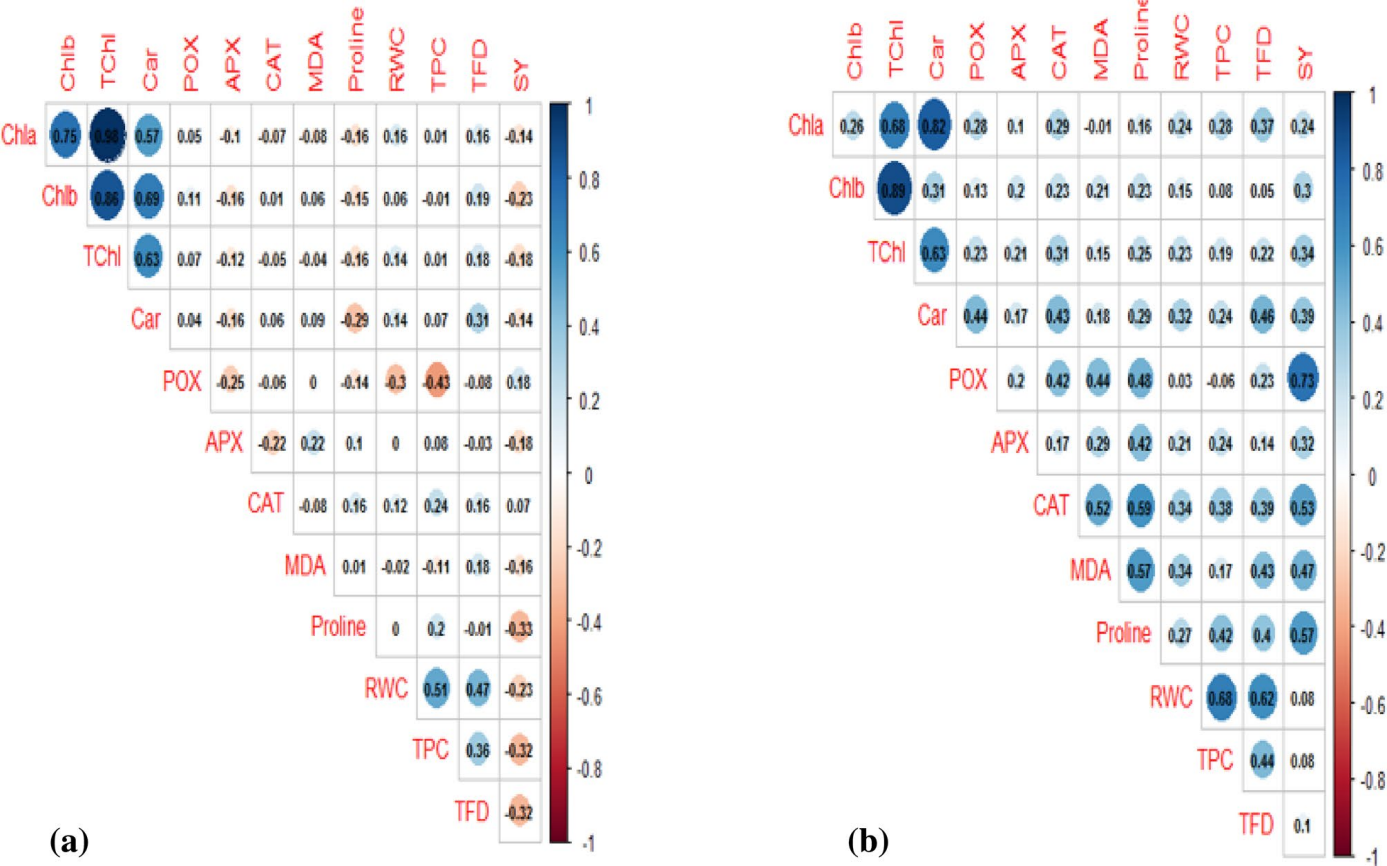
Correlation coefficients of different traits under non-stress (**a**) and drought stress (**b**) conditions averaged over the two study years (2019 and 2020).

The simple correlation coefficients were calculated for all traits under both non-stress and stress conditions (Fig. 1). Under non-stress conditions (Fig. 1a), the highest correlation coefficients were observed between TChl and Chla (0.98**), followed by TChl and Chlb (86**). The seed yield had a significant positive correlation with POX (0.18*), but a significant negative correlation with TPC (− 0.32**) and TFD (− 0.32**). Under drought-stress conditions, the highest significant positive correlation (0.89**) was observed between Chlb and TChl (Fig. 1b), followed by Chla and Car (0.82**). Seed yield had the highest significant and positive correlations with POX (0.73**), CAT (0.53**), proline (0.57**) and MDA (0.47**).

### Principal component analysis

**Figure 2:**
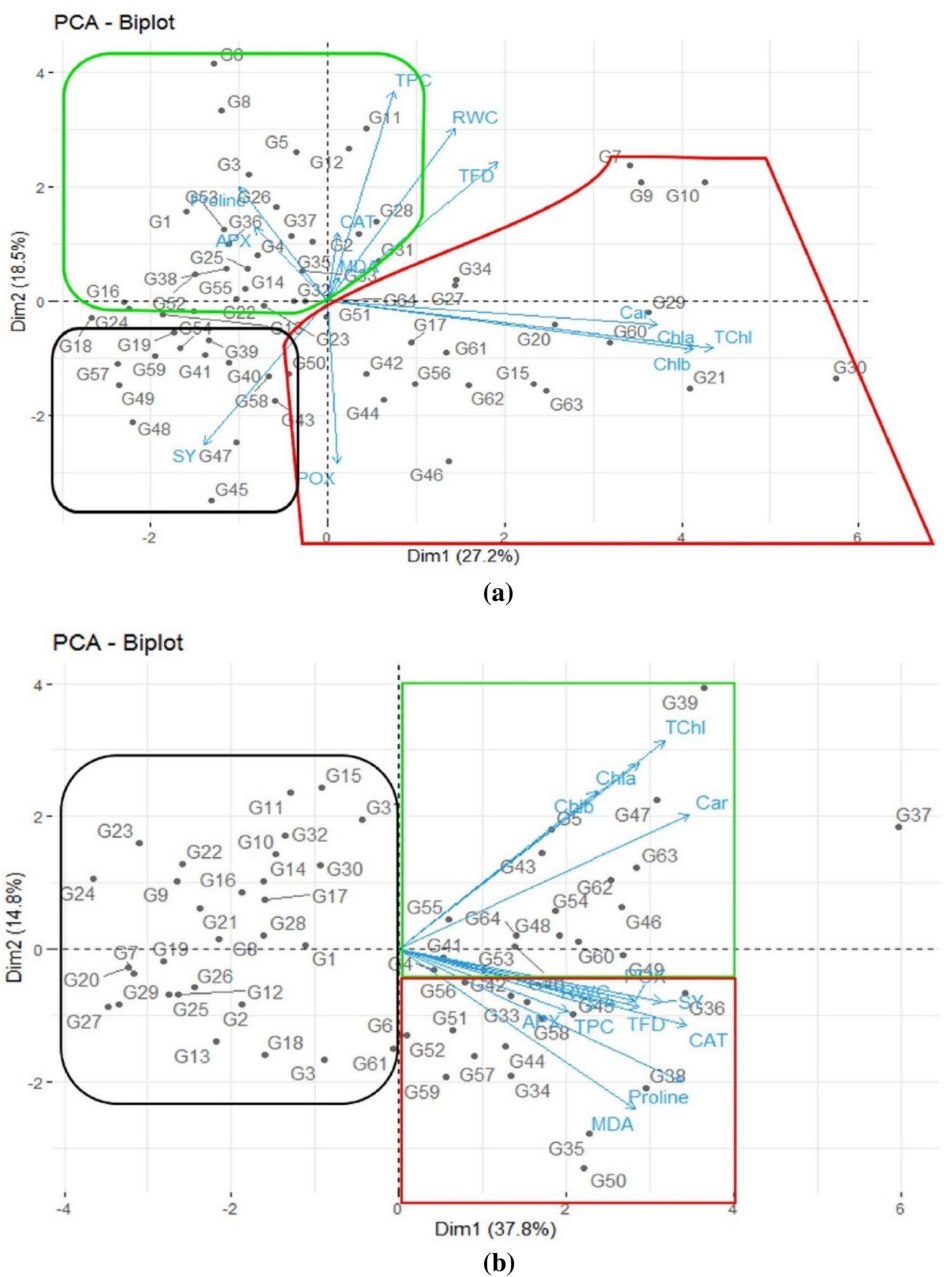
(**a**) Biplot drawn based on the first and second components obtained from principal component analysis using the seed yield and physiological traits of 64 arugula genotypes under non-stress conditions averaged over the two study years (2019 and 2020). (**b**) Biplot drawn based on the first and second components obtained from principal component analysis using the seed yield and physiological traits of 64 arugula genotypes under drought stress conditions averaged over the two study years (2019 and 2020). SY: Seed yield; Chla: Chlorophyll a; Chlb: Chlorophyll b; TChl: Total chlorophyll; Car: Carotenoids; MDA: Malondialdehyde; APX: Ascorbate peroxidase; CAT: Catalase; POX: Peroxidase; RWC: Relative water content; TPC: Total phenolics content; TFD: Total flavonoids.

Principal component analysis (PCA) was applied to distinguish the arugula genotypes with regard to their physio-biochemical and biochemical traits under the two drought stress and non-stress conditions. Accordingly, under normal conditions, the first principal component (PC_1_) explained 27.17% of the total variance in the dataset, while the second principal component (PC_2_) showed 18.53% of the variance (Fig. 2a). Under non-stress conditions, the PCA biplot identified three predominant groups mainly discriminated by their physio-biochemical traits. The first group (red group) consisted of the G_9_, G_10_, G_7_, G_34_, G_42_, G_64_, G_17_, G_46_, G_62_, G_15_, G_20_, G_60_, G_29_, G_63_, G_51_, G_44_, G_30_, G_56_, G_27_ and G_42_ genotypes. The red group genotypes in the PCA biplot had medium SY and high contents of chlorophyll, carotenoids and TFD. The second group (green group) consisted of the G_1,_ G_2_, G_3_, G_4_, G_5_, G_6_, G_8_, G_11_, G_12_, G_14_, G_25_, G_26,_ G_28_, G_32_, G_33_, G_35_, G_36_, G_37_, G_38_, G_37_, G_53_, and G_55_ genotypes. The green group genotypes in the PCA biplot had low SY, low chlorophyll and carotenoid contents, but high contents of TPC, CAT, RWC, MDA, APX and proline. The genotypes of the third group (black group) in the PCA biplot were distinguished by their high seed yield and low to moderate POX content (Fig. 2a).

The results of the second biplot explained the relationship of the traits and genotypes under drought stress. The findings showed that the first two PCs (PC_1_ and PC_2_) explained 52.52% of the total variance (Fig. 2b). The first PC (PC_1_) showed 37.76% of the total variation and had a positive correlation with all studied traits (Fig. 2b), while PC_2_ explained 14.76% of the total variance. With the exception of Chla, TChl, Car and MDA, PC_2_ showed a high positive contribution by all other studied traits (Fig. 1). The biplot identified three main groups discriminated by the traits under study. Due to having the highest TChl and Chlb content values, the G_39_ genotype was separated from the other groups. The G_5_, G_47_, G_63_, G_54_, G_55_, G_64_, G_48_, G_54_, G_62_ and G_46_ genotypes were allocated to the first group (green group), and had high accumulations of photosynthetic pigments (chlorophyll and carotenoids). The second group (red group) consisted of the G_55_, G_64_, G_46_, G_41_, G_53_, G_60_, G_36_, G_49_, G_56_, G_49_, G_42_, G_51_, G_33_, G_58,_ G_38,_ G_59_, G_57_, G_44_, G_34_ and G_35_ genotypes. These genotypes were characterized by their higher values of seed yield, RWC, TFD, TPC, POX, APX and MDA. The samples of the third group (black group) had lower values of seed yield and all other traits. The genotypes in this group had significantly lower TFD, TPC and RWC. Nevertheless, the G_50_, G_57_, G_56_, G_58_, G_45_, G_38_, G_44_ and G_49_ genotypes were identified as superior arugula genotypes that could produce the highest SY under both normal and drought stress conditions. According to the PCA results under non-stressed conditions, TChl, Chla, Chlb and carotenoids were the important components of PC_1_, while the main components of PC_2_ were proline, TPC, RWC and TFD (Table 5). However, under drought stress conditions, the important components of PC_1_ were carotenoids, CAT, proline and SY, while MDA and proline were the main components of PC_2_ (Table 5).

**Table 5:** Principal component analysis of arugula genotypes under non-stress and drought stress conditions averaged over the two study years (2019 and 2020).

Year	Non-stress		Drought stress	
Index	Component 1	Component 2	Component 1	Component 2
Chla	0.47	− 0.11	0.27	− 0.42
Chlb	0.47	− 0.11	0.22	− 0.36
TChl	0.49	− 0.11	0.30	− 0.48
Car	0.42	− 0.05	0.31	− 0.31
POX	0.01	− 0.39	0.26	0.11
APX	− 0.93	0.17	0.19	0.14
CAT	0.01	0.16	0.32	0.17
MDA	0.01	− 0.05	0.27	0.36
Proline	− 0.11	0.27	0.33	0.30
RWC	0.16	0.41	0.24	0.12
TPC	0.08	0.50	0.22	0.13
TFD	0.21	0.33	0.27	0.13
SY	− 0.15	− 0.34	0.31	0.11
Percent of variation	27.17	18.53	37.76	14.76
Cumulative percentage	27.17	45.69	37.76	52.52

*Chla* chlorophyll a, *Chlb* chlorophyll b, *TChl* total chlorophyll, *Car* carotenoids, *POX* peroxidase, *APX* ascorbate peroxidase, *CAT* catalase, *MDA* malondialdehyde, *RWC* relative water content, *TPC* total phenolics content, *TFD* total flavonoids, *SY* seed yield.

### Hierarchical cluster analysis (HCA)

**Figure 3:**
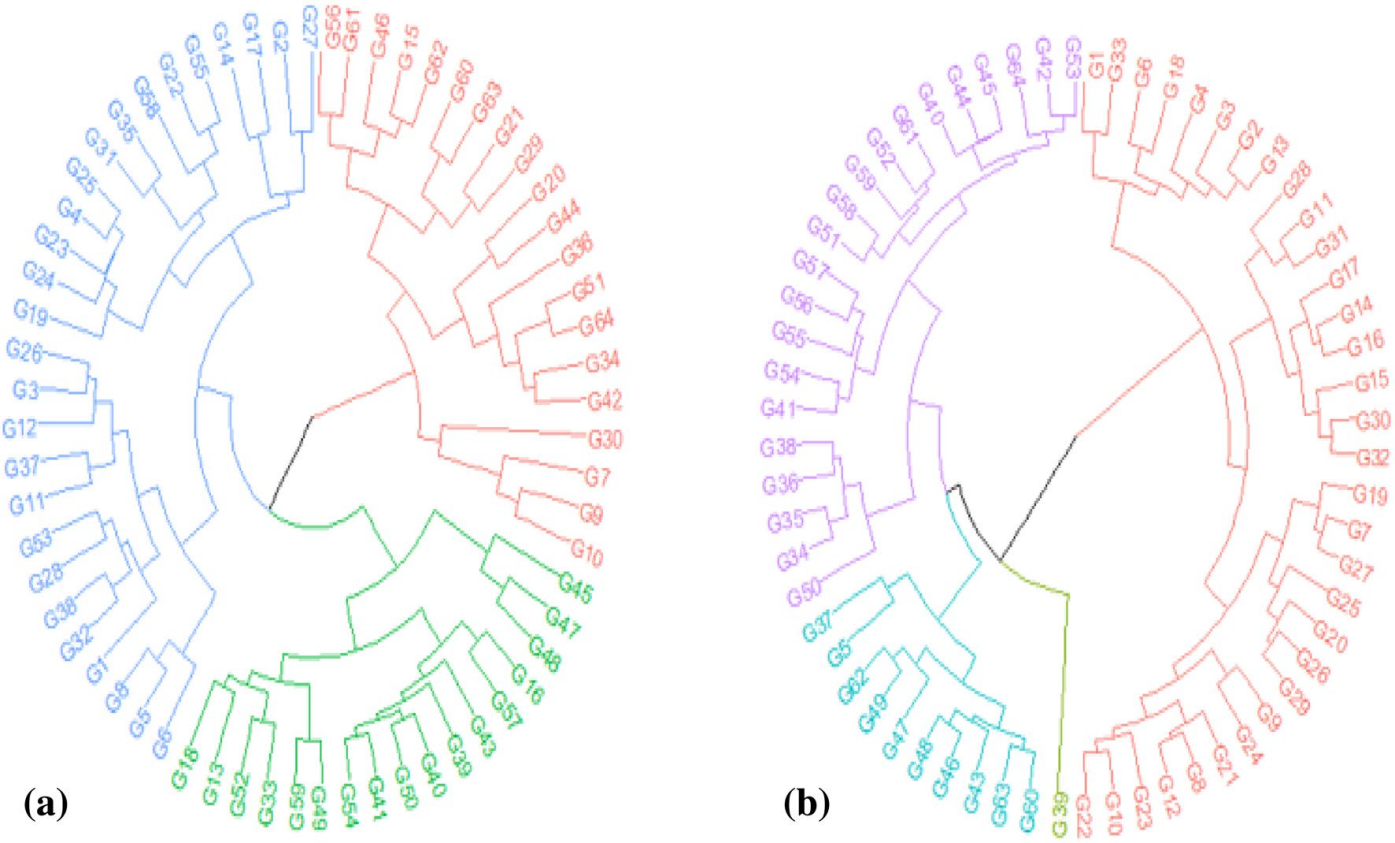
Cluster analysis using the seed yield and physiological traits of 64 arugula genotypes under non-stress (**a**) and drought stress (**b**) conditions averaged over the two study years (2019 and 2020).

To evaluate the relationships among the studied arugula genotypes, a cluster analysis was performed based on the studied traits under non-stress and drought stress conditions (Fig. 3a,b). Based on this analysis, the non-stressed arugula genotypes were classified into three distinct groups (Fig. 1a). The first group consisted of 20 genotypes: G_36_, G_64_, G_51_, G_42_, G_34_, G_44_, G_20_, G_29_, G_21_, G_63_, G_60_, G_46_, G_62_, G_15_, G_61,_ G_64,_ G_56,_ G_30_, G_10_, G_9_, and G_7_ (red color). The G_48_, G_47_, G_45_, G_16_, G_43_, G_39_, G_40_, G_50_, G_41_, G_54_, G_49_, G_59_, G_33_, G_52,_ G_13_, G_56_, and G_18_ genotypes comprised the second group (green color). The remaining 27 genotypes were allocated to the third group (blue color) (Fig. 3a).

Under drought stress conditions, the G_39_ genotype was separated from the other genotypes of the first group (Fig. 3b). The first group (blue color) consisted of 10 genotypes: G_63_, G_60_, G_48_, G_47_, G_49_, G_62_, G_46_, G_5_, G_37_, and G_43_. The G_38_, G_36_, G_35_, G_34_, G_41_, G_50_, G_54_, G_55_, G_56_, G_57_, G_51_, G_58_, G_59,_ G_52_, G_61,_ G_40,_ G_44,_ G_45_, G_64_, G_42_ and G_53_ genotypes comprised the second group (purple color)_._ The remaining 32 genotypes were allocated to the third group (red color) (Fig. 3b). Based on the hierarchical cluster analysis results, the G_39_ genotype was separated from the other genotypes of the first group due to its exceptionally high levels of Chlb and TChl under drought stress conditions. The first group had high values of chlorophyll pigments and carotenoid content, but a moderate seed yield value. The arugula genotypes of the second group (purple color) showed high values of CAT, APX, TFD, TPC, RWC and a higher value of seed yield. The remaining genotypes (red color) had low values of SY and all other studied traits (Fig. 3b, Table 4).

## Discussion

In the near future, changes in climatic conditions will result in the common occurrence of droughts, thereby posing a serious threat to food security^8^. Drought has an adverse effect on the metabolic process and seed yield of plants as it impacts their water relation, photosynthesis, and nutrient uptake^6^. The incidence of genotype × environment interaction poses difficulties for the improvement of new drought-tolerant genotypes in crops. Therefore, the selection of drought-tolerant genotypes, and the elucidation of the underlying mechanisms by plant-breeding scientists are both essential to the increase of agricultural production in arid and semi-arid regions. The findings of this study showed a highly diversified array of genotypic responses to both non-stress and drought stress conditions with regard to all studied traits. The genotype comparisons under drought stress and non-stress conditions showed that certain genotypes had a cross-over genotype × environment interaction (G × E). This means that some genotypes are appropriate for typical environments, but not suitable for water-deficit conditions. For example, the SY values were 162.33 g^−1^ plant and 178 g^−1^ plant in the G_14_ and G_18_ genotypes under non-stress conditions, respectively (Table 3), but their SY under stress conditions were 99.3 g^−1^ plant and 97.1 g^−1^ plant, respectively (Table 4). The data for SY suggested that the G_50_ and G_57_ genotypes were the most drought-tolerant (Table 4). Therefore, drought-tolerant genotypes can be considered as superior and seem to be suitable candidates for breeding programs in drought-stressed environments. The decrease in SY under drought stress can be related to the reduction of photosynthetic activities, due to the stomatal closure and limited carbon dioxide uptake^17^.

Moreover, lipid peroxidation was induced by apoplastic ROS, making MDA a reliable marker for membrane damage, particularly under drought conditions^9^. Therefore, under environmental stresses, MDA may have certain damaging impacts on plants, such as the interruption of photosynthetic pigments, inhibition of enzyme activity, protein denaturation, and programmed cell death^18^. Similarly, a number of previous studies reported enhanced levels of MDA in other plant species under drought stress conditions^19,20^. According to the findings of these studies, drought-induced oxidative stress was moderate to severe at the flowering stage of these genotypes.

Under drought stress, the RWC serves as a marker for the leaf water status. The RWC may also indicate the balance between the water absorbed by the plant and the water consumed through transpiration. In this study, a considerable decrease in RWC was observed in all genotypes. However, the amount of RWC loss varied among different genotypes. The results of this research showed a non-significant correlation between SY and RWC (0.08) under drought stress, which were different from the findings of other studies conducted on other species, such as safflower^21^ and wheat^22^. These dissimilarities could be due to the differences in stress duration, drought intensity, genotype, and species^23^. This may be because, rather than conserving water in their leaves, the arugula genotypes apply other defensive mechanisms to tolerate drought. Therefore, RWC cannot be considered as a good selective biochemical marker for drought-tolerance in these arugula genotypes. The reduction of RWC under drought was greater in G_24_ (53.02%), which showed the water status in this genotype to be more susceptible to drought than the other genotypes. On the other hand, G_37_ and G_38_ were considered the best genotypes for conserving water content under drought stress conditions.

Photosynthetic pigments (such as carotenoids and chlorophylls) play a crucial role in photosynthesis, as they protect the photosynthetic apparatus from the harmful impacts of ROS under drought stress^24^. Carotenoids have a significant role in hindering oxidative damage by quenching chlorophyll triplets and singlet oxygen^25^. The decrease in photosynthetic pigments in the drought-stressed arugula genotypes may be due to certain drought-induced events. For example, the rapid decomposition of chlorophyll could subsequently decrease the carbon dioxide exchange rate and damage the chloroplast structures^24,26^. The chlorophyll and carotenoid content decrease in these genotypes was similar to those reported in safflower^19,21^, fennel^17^, canola^27^, and *Amaranthus tricolor*^28^. The reduction in photosynthetic pigments can directly limit photosynthetic potential, and consequently reduce seed production^17^. This confirms the positive correlation of SY with Chla (r = 0.29**), TChl (0.31**) and carotenoids (r = 0.41**) under drought stress (Fig. 1b). In this regard, our data highlighted a noteworthy photosynthetic performance of the G_24_ genotype under drought stress.

Proline plays a vital role in stabilizing sub-cellular structures and increasing drought stress tolerance through osmotic pressure adjustment^29,30^. Therefore, greater accumulations of proline are linked to increased stress tolerance. As a molecular chaperone, proline acts as an antioxidative defense molecule. Proline scavenges reactive oxygen species (ROS) to activate specific gene functions that are crucial for the plant’s recovery from stresses^30^. In certain industrial crops, such as safflower^19,21^, fennel^17^, cotton^31^, and maize^32^, an enhanced level of proline (as an osmolyte metabolite) has been reported to be a marked response to drought stress. In cases as such, the increase in proline content under drought could be due to the induction of proline biosynthesis, as well as the inhibition of its oxidation under drought stress^30^. In this study, the significant correlation between proline and SY (0.56**) demonstrated the important adjusting role of this osmolyte under drought stress in the arugula plant. Also, the positive correlation of proline with POX (0.47**), APX (0.42**) and CAT (0.59**) suggested the effective role of proline in maintaining cell turgidity under drought, and the subsequent enhancement of antioxidant enzyme activity (POX, APX and CAT)^30,32^. Under drought stress, the correlation between the phenolic traits (TFD and TPC) and proline was non-significant. This can be due to the independent biosynthetic pathways of the phenolic compounds (shikimate/phenylpropanoid pathway)^33^ and proline (glutamate pathway) in this genus.

Plant enzymes such as APX, POX and CAT are able to scavenge H_2_O_2_ with different mechanisms and suppress its toxic effects^34^. The scavenging of H_2_O_2_ production under drought stress conditions is a common phenomenon, which is done by both the enzymatic (POX, CAT, and APX) and non-enzymatic antioxidants^35^. In this study, the drought stress caused an increase in enzymatic activity (CAT, POX and APX) (Table 2), which was in agreement with the results of other reports^13,21,36,37^. Another study reported the efficient role of APX in scavenging H_2_O_2_ in the ascorbate–glutathione cycle of plant cells under drought stress conditions^34^. The significant positive correlation of the studied enzymatic antioxidants (APX, POX and CAT) with SY, and the positive correlation of MDA with CAT (0.52**), POX (0.29*) and APX (0.44**) under drought stress, may be due to the adequate effects of these antioxidants, as they can scavenge the ROS species and alleviate the harmful effects of the drought stress. These results are similar to the findings of previous reports on other species^21,28,38^. Therefore, these enzymatic antioxidants (POX, APX and CAT) can be used as efficient and economical biochemical indices to either screen or enrich arugula germplasm for drought tolerance in the early growth stages. Catalase is considered as a key ancillary component of photosynthesis in green leaves by preventing ROS accumulation^39^. When plants are subjected to drought stress, the maintenance and subsequent increase of CAT activity in the plant leaves can result in the removal of the produced photo respiratory H_2_O_2_. This confirms the significant increase of CAT activity under drought stress in the current study. The significant increase of CAT activity appears to be linked to lower oxidative damage, which was previously detected in plants with increased CAT activity.

Moreover, CAT activity had a positive correlation with RWC (0.34**), which was similar to reports by Alizadeh Yeloojeh et al.^21^ on safflower and Merwad et al.^40^ on cowpea (*Vigna unguiculata*). An association between increased CAT activity and greater water retention in the arugula leaves was observed. Therefore, genotypes that maintain increased CAT activity under drought stress in their leaves may show a greater water retention ability and higher stress tolerance.

Phenolic compounds have antioxidant characteristics and scavenge the free radicals generated under drought stress^41^. The positive correlations of RWC with TFD (0.62*) and TPC (0.68**) under drought stress and non-stress conditions (Fig. 1) demonstrate the positive effects of TFC and TPC as secondary metabolites in maintaining the chloroplast content and turgor pressure of both non-stressed and stressed arugula leaves^41^. When compared to non-stressed conditions, the non-significant changes in TPC and TFD under drought stress may be due to the involving effects of other factors, such as the degradation of photosynthetic pigments, which play an important role in their synthesis^42^. Therefore, it may be concluded that polyphenolics act as subtracts for the synthesis of enzymes in the antioxidant defense network^43^. The second hypothesis suggests that plants, instead of using the phenolic compounds, primarily attempt to reduce their ROS levels with antioxidative enzymes^43^.

The arugula genotypes mostly showed a similar stress response pattern, which included the accumulation of proline, oxidative damage to membrane lipids, elevation in hydrogen peroxide content, increase of the POX, APX, and CAT antioxidant activities and proline content, and the reduction of photosynthetic pigments (carotenoids and chlorophyll). Distinct regulation mechanisms were observed under drought-induced oxidative stress by the various trends of these enzymatic and non-enzymatic antioxidants.

The results of the clustering analysis were moderately consistent with the identifications of the three groups in PCA. Under normal irrigation conditions, the cluster analysis and PCA allocated 49% of the genotypes to the same groups. However, under drought stress conditions, the cluster analysis and PCA placed approximately 86% of the genotypes in the same groups. The hybridization between the first and third group genotypes that had the greatest distance under stress conditions, could lead to the production of hybrids with increased secondary metabolites and heightened enzyme activities, which is highly beneficial to the development of arugula in dry areas.

## Conclusion

According to our observations on a global collection of arugula genotypes, the drought stress caused a broad range of variation in seed yield and various other physio-biochemical traits, such as the phenolic compounds, enzymatic antioxidants, RWC, and MDA content. Based on the current results, the highly active enzymatic antioxidants APX, CAT, and POX were responsible for the higher drought-tolerance in arugula genotypes. Based on the principal component analysis, the G_50_, G_57_, G_54_, G_55_ and G_60_ genotypes could produce higher seed yields under drought stress conditions. This new information may be used to breed drought-tolerant genotypes by selecting superior genotypes of arugula. Subsequent researches should be conducted in different geographical regions and under different environmental stress conditions to confirm the superiority of genotypes that contain valuable genes.

## Materials and methods

### Ethics statement

The plant seeds were collected and handled in accordance with all relevant guidelines.

### Plant materials and Experimental design

The seeds of the non-Iranian accessions were collected from the Leibniz Institute of Plant Genetics and Crop Plant Research (IPK), Germany. After considering the descriptions of each genotype (Table S1), the genotypes were planted based on a completely randomized block design with three replications under both non-stress and drought conditions from October to June of 2018–2019 and 2019–2020 at Isfahan University of Technology, Iran (32° 32′ N, 51° 23′ E, 1630 asl). Each plot, as an experimental unit, consisted of two rows that were 2 m in length and 70 cm apart. The plants were spaced 10 cm apart within the rows. The soil was characterized as silty clay loam with a bulk density of 1.3 g cm^−3^ and a pH range of 7.4–7.9. The monthly temperature (mean, max, and min) and rainfall values are shown in Fig. S1a,b. The reported mean annual temperatures and precipitations were, respectively, 19.4 °C and 98.6 mm in 2019, and 19.41 °C and 159 mm in 2020.

### Drought stress assay

The drought stress treatments were applied as described by Nikzad et al.^44^. The irrigation depth was calculated using the following formula: I = [(FC − *θ*)/100] D × B); where I is irrigation depth (cm), FC (− 0.03 MPa) is soil gravimetric moisture percentage at field capacity (22%), *θ* (− 1.5 MPa) is soil gravimetric moisture percentage at irrigation time (10%), D is root-zone depth (50 cm), and B is soil bulk density at the root zone (1.3 g cm^−3^)^45^. Based on the plant’s water requirements, the non-stress and drought stress treatments were irrigated uniformly and simultaneously from the beginning of the trial until the initial stages of flowering (10%). Later, the non-stress and drought stress treatments were irrigated after 50% and 80% of water drainage, respectively. A pressurized irrigation system using polyethylene drip-irrigation tapes (16 mm diameter) was applied beside each planting row. In each plot, the mature and young apical leaves of the plants (from the middle of each row) were selected at the maturity stage to measure the biochemical traits. The leaf samples were then frozen and stored at − 80 °C for further analysis. Sampling was done using 10 randomly selected plants from each treatment. Finally, the plants were harvested after edge effect removal and the grain yields were determined accordingly.

### RWC measurement (%)

The following equation was used for determining the relative water content (RWC): RWC (%) = [(FW–DW)/(TW–DW)] × 100^17^. Turgid weight (TW) was determined after the imbibition of the leaves in distilled water for 4 h. Dry weight (DW) was determined after the incubation of the leaves in an oven at 80 °C for two days.

### Proline assay

To perform the proline assay^46^, 3 ml of sulfosalicylic acid (3% w/v) was added to the leaf samples (0.2 g). The mixture was then centrifuged at 18,000× *g* for 15 min. The supernatant (2 ml) was then put into a new tube and 2 ml of glacial acetic acid and 2 ml of the ninhydrin reagent were added to the test tubes. The tubes were immersed in a 100 °C hot water bath for an hour, after which the solution was cooled immediately on ice. Toluene (4 ml) was added into the mixture to stop the reaction. The toluene phase absorbance was measured at 520 nm using a spectrophotometer.

### Chlorophyll and carotenoids assay

The chlorophyll a (Chla), chlorophyll b (Chlb), total chlorophyll (TChl), and carotenoid (Car) contents were assayed according to methods described by Lichtenthaler^47^. The first step was to squash fresh leaf samples (0.2 g) in a mortar by adding 10 ml of acetone 80% (Merck, Com.) until they were completely colorless. The solutions were then centrifuged at 6000× *g* for 10 min. A spectrophotometer was used to record the absorption value of the leaf extract solution at 662 nm for Chla, at 645 nm for Chlb, and at 470 nm for carotenoids using a UV–Vis spectrophotometer (UV-1800, Shimadzu). The results were expressed as mg of pigment per gram of leaf fresh weight.

### Malondialdehyde assay

To measure the malondialdehyde (MDA) content^19^, powdered leaf samples (0.5 g) were mixed with 5 ml of potassium phosphate buffer (50 mM, pH = 7). After centrifuging the homogenate at 15,000× *g* for 10 min at 4 °C, 1.5 ml of thiobarbituric acid (TBA) 0.5% in trichloroacetic acid (TCA) 20% (w/v) was added to 0.5 ml of the supernatant. The mixture was first heated at 95 °C for 30 min and then kept in ice for 30 min to stop the reaction. Finally, the absorption value was measured at 532 nm and 600 nm for the correction of nonspecific turbidity. The MDA content was expressed as nanomol g^−1^ FW.

### Extract preparation for antioxidant enzymes assay

Fresh leaf samples (0.2 g) of each treatment were ground using a mortar and pestle. Afterwards, a 2 ml sodium-phosphate buffer (10 mM, pH = 7) that included 50 mM potassium phosphate, 1 mM ethylene diamine tetra acetic acid (EDTA), 2% polyvinyl pyrrolidone, 2 mM α-dithiothreitol (DTT), 50 mM Tris HCl, 0.2% triton x-100 was added to the samples. The mixture was centrifuged at 12,000× *g* for 30 min. The supernatant was collected for antioxidant enzyme evaluation.

### Catalase assay

Hydrogen peroxide (H_2_O_2_) consumption was utilized to measure the catalase activity (CAT) spectrophotometrically at 240 nm^48^. The assay buffer included 50 ml of enzyme extract, 15 mM of H_2_O_2_, and 50 mM of potassium phosphate buffer (pH 7.0).

### Ascorbate peroxidase assay

The ascorbate peroxidase (APX) assay buffer contained H_2_O_2_ (0.5 mM), enzyme extract (50 ml), potassium phosphate buffer (50 mM) (pH 7.0), and ascorbate (5 mM)^49^. Based on the reduction in absorbance, the APX activity was measured spectrophotometrically at 265 nm.

### Peroxidase assay

After making minor modifications, the peroxidase (POX) activity was measured specifically with guaiacol^49^. The increase in absorbance at 470 nm was recorded in a mixture of 0.1 ml of enzyme extract with 3 ml of phosphate buffer (50 mM; pH = 7) containing 0.05 ml guaiacol and 0.03 ml H_2_O_2_. Enzyme activity was expressed as units enzyme activity per mg of protein.

### Total phenolic and flavonoid content

To prepare a methanolic extract of the arugula genotypes, 250 mg of powdered plant was extracted with 10 ml of 80% methanol by slow shaking. The obtained solution was filtered and maintained for further analysis.

To obtain the total phenolic content (TPC), 2.5 ml of the Folin–Ciocalteu reagent (1:10 diluted with distilled water) and 2 ml of 7.5% sodium carbonate solution were added to 0.5 ml of the methanolic extract according to Priyanthi and Sivakanesan^50^. The mixture was incubated at 45 °C for 15 min. Finally, the absorbance of the solution was read at 765 nm using a spectrophotometer. The TPC value was expressed as mg of tannic acid equivalent (TAE) per gram of each extract on a dry basis.

To estimate the total flavonoid content (TFD), the diluted extract (125 µl) was mixed with 300 µl of 5% NaNO_2_ solution and incubated for 5 min^19^. The mixture was then blended with 600 µl AlCl_3_ (10% w/v). In the end, the blend was mixed with 2000 µl of NaOH (1 M) and 2000 µl distilled water and used to reach the final volume of the solution. The observations were recorded at 510 nm. The TFD content was expressed as mg of quercetin equivalents (QE) per gram of each extract on a dry basis.

### Statistical analyses

The combined data from the two years of study were subjected to analysis of variance (ANOVA) using the SAS statistical software (ver. 9.4, SAS Institute Inc., Cary, NC, USA). Mean comparisons were conducted using Fisher’s least significant difference (LSD) test at *P* < 0.05. Correlation analysis and principle component analysis (PCA) were done using the R-software (ver. 3.4.3). Cluster analysis was conducted using Ward’s method based on linkage distances with Stat graphics Centurion ver. 18.1.12.

## Supplementary Information

Supplementary Information.

## Data Availability

The datasets used and/or analyzed during the current study are available from the corresponding author upon reasonable request.
