# Supplementary material for: Response of seed yield and biochemical traits of Eruca sativa Mill. to drought stress in a collection study
Source: Sci Rep. 2023 Jul 10;13:11157. doi: 10.1038/s41598-023-38028-6 (PMC10333284; doi:10.1038/s41598-023-38028-6)
Supplement: Supplementary file 1 — Supplementary Information. [file 41598_2023_38028_MOESM1_ESM.docx]

| **Table S1.** Geographical origins and genotypic codes of the 64 *E. sativa* Mill. accessions. | | | | |
| --- | --- | --- | --- | --- |
| Origin | Genotype code | Origin | Genotype code | |
| Egypt (Egypt2) | G_33_ | Kashan, Kashan, Iran (Iran1) | G_1_ |  |
| Italy (Italy1) | G_34_ | Natanz, Isfahan, Iran (Iran2) | G_2_ |  |
| Italy (Italy2) | G_35_ | Ardestan, Isfahan, Iran (Iran3) | G_3_ |  |
| Italy (Italy3) | G_36_ | Isfahan3, Isfahan, Iran (Iran4) | G_4_ |  |
| Italy (Italy4) | G_37_ | Isfahan2, Isfahan, Iran (Iran5) | G_5_ |  |
| Italy (Italy5) | G_38_ | Shiraz, Shiraz Iran (Iran6) | G_6_ |  |
| Italy (Italy6) | G_39_ | Ashkezar, Yazd, Iran (Iran7) | G_7_ |  |
| Italy (Italy7) | G_40_ | Kerman, Kerman, Iran (Iran8) | G_8_ |  |
| Italy (Italy8) | G_41_ | Khozestan, Khozestan, Iran (Iran11) | G_9_ |  |
| Italy (Italy9) | G_42_ | TaLkhoncheh, Isfahan, Iran (Iran12) | G_10_ |  |
| Italy (Italy10) | G_43_ | Sarbishe, Khorasan, Iran (Iran13) | G_11_ |  |
| Italy (Italy11) | G_44_ | Afghanistan (Afghanistan1) | G_12_ |  |
| Italy (Italy12) | G_45_ | Afghanistan (Afghanistan2) | G_13_ |  |
| Italy (Italy13) | G_46_ | Afghanistan (Afghanistan3) | G_14_ |  |
| Spain (Spain1) | G_47_ | Pakistan (Pakistan1) | G_15_ |  |
| Spain (Spain2) | G_48_ | Pakistan (Pakistan2) | G_16_ |  |
| Germany (Germany1) | G_49_ | Pakistan (Pakistan3) | G_17_ |  |
| Germany (Germany2) | G_50_ | Pakistan (Pakistan4) | G_18_ |  |
| Germany (Germany3) | G_51_ | Pakistan (Pakistan5) | G_19_ |  |
| Russia (Russia1) | G_52_ | Pakistan (Pakistan6) | G_20_ |  |
| Russia (Russia2) | G_53_ | Pakistan (Pakistan7) | G_21_ |  |
| Russia (Russia3) | G_54_ | Pakistan (Pakistan8) | G_22_ |  |
| Russia (Russia4) | G_55_ | Pakistan (Pakistan9) | G_23_ |  |
| Turkey (Turkey1) | G_56_ | Jordan (Jordan1) | G_24_ |  |
| Turkey (Turkey2) | G_57_ | Jordan (Jordan2) | G_25_ |  |
| Belgium (Belgium) | G_58_ | Syria (Syria1) | G_26_ |  |
| Croatia (Croatia1) | G_59_ | Syria (Syria2) | G_27_ |  |
| Croatia (Croatia2) | G_60_ | China (China) | G_28_ |  |
| Portugal (Portugal) | G_61_ | Libya (Libya1) | G_29_ |  |
| Netherland (Netherland) | G_62_ | Libya (Libya2) | G_30_ |  |
| Poland (Poland) | G_63_ | Libya (Libya3) | G_31_ |  |
| Austria (Austria) | G_64_ | Egypt (Egypt1) | G_32_ |  |

**Figure S1.** **(a)** Monthly mean temperature (°C ) during different seasons averaged over 2019-2020; **(b)** Monthly rainfall ( mm) during different seasons averaged over 2019-2020.
